# Oncogenic RAS sensitizes cells to drug-induced replication stress via transcriptional silencing of P53

**DOI:** 10.1038/s41388-022-02291-0

**Published:** 2022-04-07

**Authors:** Hendrika A. Segeren, Elsbeth A. van Liere, Frank M. Riemers, Alain de Bruin, Bart Westendorp

**Affiliations:** 1 Department of Biomolecular Health Sciences, Faculty of Veterinary Medicine, Utrecht University, Utrecht, The Netherlands; 2 Department of Clinical Sciences, Faculty of Veterinary Medicine, Utrecht University, Utrecht, The Netherlands; 3 Department of Pediatrics, University of Groningen, University Medical Center Groningen, Groningen, The Netherlands

**Keywords:** Chemotherapy, Tumour heterogeneity, DNA replication, Checkpoint signalling, Growth factor signalling

## Abstract

Cancer cells often experience high basal levels of DNA replication stress (RS), for example due to hyperactivation of oncoproteins like MYC or RAS. Therefore, cancer cells are considered to be sensitive to drugs that exacerbate the level of RS or block the intra S-phase checkpoint. Consequently, RS-inducing drugs including ATR and CHK1 inhibitors are used or evaluated as anti-cancer therapies. However, drug resistance and lack of biomarkers predicting therapeutic efficacy limit efficient use. This raises the question what determines sensitivity of individual cancer cells to RS. Here, we report that oncogenic RAS does not only enhance the sensitivity to ATR/CHK1 inhibitors by directly causing RS. Instead, we observed that HRAS^G12V^ dampens the activation of the P53-dependent transcriptional response to drug-induced RS, which in turn confers sensitivity to RS. We demonstrate that inducible expression of HRAS^G12V^ sensitized cells to ATR and CHK1 inhibitors. Using RNA-sequencing of FACS-sorted cells we discovered that P53 signaling is the sole transcriptional response to RS. However, oncogenic RAS attenuates the transcription of P53 and TGF-β pathway components which consequently dampens P53 target gene expression. Accordingly, live cell imaging showed that HRAS^G12V^ exacerbates RS in S/G2-phase, which could be rescued by stabilization of P53. Thus, our results demonstrate that transcriptional control of P53 target genes is the prime determinant in the response to ATR/CHK1 inhibitors and show that hyperactivation of the MAPK pathway impedes this response. Our findings suggest that the level of oncogenic MAPK signaling could predict sensitivity to intra-S-phase checkpoint inhibition in cancers with intact P53.

## Introduction

Preservation of genomic integrity is essential for life and therefore strictly controlled by DNA damage checkpoints. However, excessive activity of oncoproteins such as MYC, RAS and Cyclin E pose a threat to faithful propagation of DNA by induction of endogenous replication stress (RS) [1, 2]. RS is referred to as impediments during DNA replication and thus includes stalling and collapsing of replication forks. Emerging evidence uncovers mild endogenous RS in the vast majority of cancer cells and is therefore considered a hallmark of cancer [3].

Activation of the intra S-phase checkpoint is the key response to RS and initiated by the binding of Replication Protein A (RPA) to single-stranded DNA that is exposed when replication fork progression hampers. RPA recruits, with the aid of ATR-interacting protein (ATRIP), Ataxia Telangiectasia and Rad3-related protein (ATR) to the stressed fork. Upon mild RS, a wide range of proteins, such as RPA2 and 53BP1, are recruited and phosphorylated to facilitate fork stabilization [4–7]. When severe RS is induced, ATR activates Checkpoint Kinase 1 (CHK1) and changes the focus to fork repair and the delay of cell cycle progression [6–8]. The ATR-CHK1 axis couples faithful DNA replication to cell cycle progression by inhibiting CDK activity. In detail, CHK1 targets the CDK-activating protein CDC25a for degradation and activates the CDK-inhibitor WEE1 [6, 8]. WEE1 -together with P53- is also of crucial importance to enforce the G2/M checkpoint, which prevents mitotic entry by inhibiting CDK1 activity [9]. Together, the intra S-phase checkpoint and G2/M checkpoint are of critical importance to ensure that cells only enter mitosis with a fully replicated and undamaged set of genetic information. When RS is not repaired and cell cycle progression continues, cells undergo excessive DNA damage, also known as replication catastrophe, and cell death [10–12].

Since high levels of RS are detrimental for survival, cancer cells heavily rely on the intra S-phase checkpoint [13–15]. Accordingly, an extra allele of CHK1 protects against oncogene-induced RS [16] and high levels of CHK1 confer resistance to RS-inducing drugs [17]. The dependency of cancer cells on the intra S-phase checkpoint has led to the development of ATR and CHK1 inhibitors (reviewed in [18]). These inhibitors are combined with classical chemotherapeutic drugs to completely exhaust the intra S-phase checkpoint and together referred to as RS-inducing drugs. However, the lack of knowledge on parameters that predict sensitivity to these drugs limits efficient clinical use, while drug resistance remains a major problem [19]. In contrast, selection of BRCA mutant tumors for PARP inhibitor treatment shows that patient selection can dramatically improve drug efficiency. Interestingly, the lack of P53 can sensitize cells to ATR and CHK1 inhibition [20], suggesting a potential selection criterium for treatment with RS-inducing drugs. Nonetheless, P53 mutation status failed to predict the response to inhibitors of the intra S-phase checkpoint in xenograft experiments and clinical trials [21–23].

Besides P53, little focus has been paid to the role of oncogenic alterations in the response to RS-inducing drugs. Hyperactivation of the MAPK pathway by mutations in one of the RAS isoforms is one of the most frequent alterations in human malignancies [24]. Oncogenic RAS elevates reactive oxygen species (ROS) and increases global transcription rates leading to high basal levels of RS [25, 26]. As a result, RAS-mutant cells heavily rely on the intra S-phase checkpoint for survival [13, 15] and tumors with oncogenic RAS are promising candidates for treatment with RS-inducing drugs. Interestingly, conflicting studies showed that RAS could either increase or reduce P53 levels [27, 28]. More recently, ERK signaling, downstream of RAS, was shown to moderate the pulsatile behavior of P53 signaling, which permits proliferation in the presence of mild damage [29]. However, despite the key role of ERK signaling in the proliferation-quiescence decision [30], the effect of oncogenic RAS on cell fate decisions upon intra S-phase checkpoint inhibition has been neglected.

In addition to the variety of mutations a tumor can harbor, cell-intrinsic heterogeneity in cell cycle phase and level of RS blur the picture when evaluating the response to RS-inducing drugs. Therefore, it is of critical importance to firstly determine how a single cell responds to RS-inducing drugs and secondly evaluate how mutations affect this process to optimize anti-cancer treatment with RS-inducing drugs.

Here we use non-transformed human cells containing fluorescent cell cycle and RS reporters to show that regulation of P53 target genes is essential to control the response to RS. We demonstrate that inducible HRAS^G12V^ impinges the RS response by transcriptional downregulation of P53 and TGF-β signaling, resulting in downregulation of DNA repair genes in S/G2-phase. Suppression of the P53-dependent gene transcription program by oncogenic HRAS sensitizes cancer cells to RS-inducing drugs, and decreases long-term viability after transient treatment with these drugs.

## Results

### Development of an in vitro model with multiple reporters to study the RS-response in living single cells

**Fig. 1:**
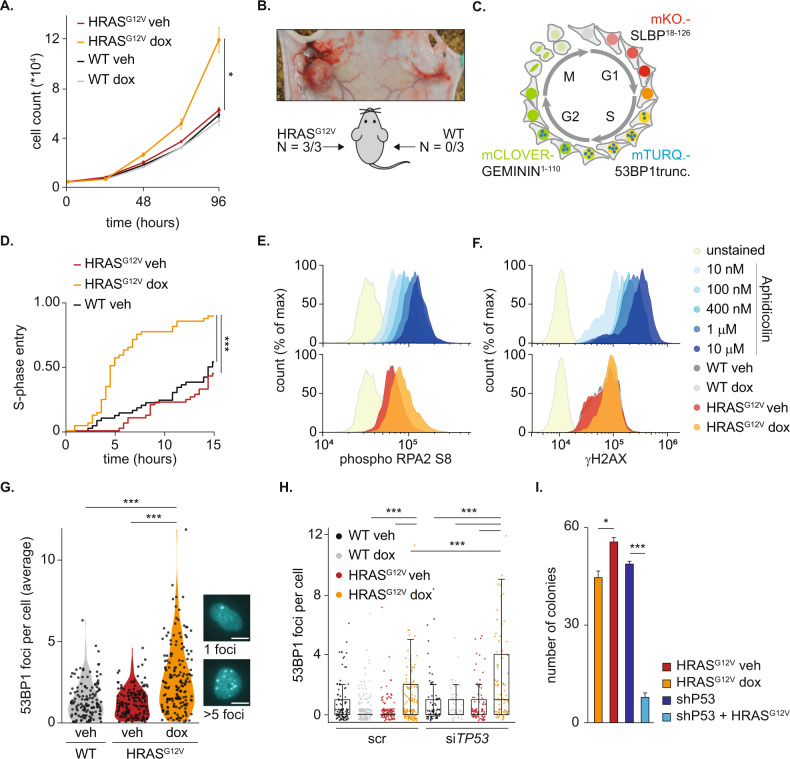
Inducible HRAS^G12V^ shortens G1-phase and induces mild RS. **A** Cell proliferation, as measured by cell counting, of RPE WT or HRAS^G12V^ cells with or without doxycycline. Measurement indicates mean ± s.e.m., statistical analysis was performed with a Kruskal–Wallis test. **B** Representative image and quantification of RPE WT and HRAS^G12V^ cells injected in mice. This resulted in tumor formation in cells expressing HRAS^G12V^ (left). **C** Schematic representation of FUCCI4 system and fluorescent tagged 53BP1 during cell cycle progression. **D** Cumulative frequency plot of WT and HRAS^G12V^ cells with or without doxycycline which were in G1-phase at the start of imaging. The time until S-phase entry of at least 25 individual cells per condition was measured. Differences were statistically evaluated with a Log-rank test. **E** Representative example of flow cytometry data of phospho RPA2 S8 staining. Only S-phase cells were selected, based on DNA content, for analysis. Cells were collected after 24 h treatment with doxycycline or 16 h treatment with Aphidicolin. **F** Same as in E, but cells were now stained with an antibody against γH2AX. **G** Violin plot showing the number of 53BP1 foci, as read out for RS. Average number of foci in S-phase per frame in the absence or presence of 24 h oncogenic RAS induction is shown. 100 cells per condition were analyzed. Statistical differences were evaluated using a Kruskal–Wallis test and post-hoc Dunnett’s test. Scale bar represents 10 μm. **H** Dot plot showing the number of 53BP1 foci in individual S-phase cells 24 h after siRNA and doxycycline treatment for indicated conditions analyzed from pictures of RPE FUCCI4 cells. Per condition at least 50 cells were evaluated. Differences were statistically tested using a Kruskal–Wallis test with post-hoc Dunnett’s test with Benjamin Hochberg correction. **I** Quantification of colony formation assay with indicated cells. Bars represent mean ± s.e.m. Statistical differences were evaluated using a Kruskal–Wallis test and post-hoc Dunnett’s test.

The response of cancer cells to RS is most likely affected by the combined action of multiple oncogenic changes. This makes it complicated to address the effect of single mutations. We therefore utilized human non-transformed hTERT RPE-1 cells in which we introduced oncogenic HRAS (HRAS^G12V^) under the control of a Tet repressor. Upon administration of doxycycline, these cells hyperactivated the MAPK signaling pathway (Fig. S1A–C). In accordance with this oncogenic change, HRAS^G12V^ stimulated cell proliferation (Fig. 1A). Remarkably, induction of HRAS^G12V^ was sufficient to allow transformation and subcutaneous tumor growth of RPE cells in mice (Fig. 1B).

We then investigated which phases of the cell cycle were accelerated by HRAS^G12V^. Therefore, we introduced the Fluorescent Ubiquitination-based Cell Cycle Indicator (FUCCI) 4 system [31] into these cells (Fig. 1C). Live cell imaging revealed that the increased proliferation speed of cells with HRAS^G12V^ could be attributed to a shorter G1-phase, while length of other phases was unaffected (Fig. 1D and Fig. S1D). In accordance with previously published data [32–34], we identified in G1 cells with oncogenic RAS elevated levels of *CCND1*, downregulation of the CDK inhibitors *CDKN1A* and *CDKN1B*, and repression of TGF-B pathway components *TGF-B1* and *SMAD3* (Fig. S1E–G). Together, this can promote CDK activity and consequently stimulate S-phase entry which results in a shortened G1-phase in cells expressing HRAS^G12V^.

A shortened G1-phase can directly induce RS by at least two distinct mechanisms. Firstly, premature S-phase entry prevents transcription-mediated silencing of replication origins in G1, resulting in deregulated origin firing [35]. Replication forks of these origins are prone to collapse and thereby contribute to oncogene-induced RS [35]. Secondly, unscheduled cell cycle entry results in co-occurrence of transcription and replication events, which are normally separated in different cell cycle phases [36]. In addition, oncogenic RAS elevates global transcription rates [26]. The increased transcription rate in S-phase results in collisions between the replication and transcription machinery and formation of secondary nucleotide structures which impede faithful DNA replication [35]. To evaluate if HRAS^G12V^ also caused RS in RPE cells we performed DNA fiber assays. In this assay cells are pulsed with fluorescent labelled nucleotides for a set amount of time, from which origin firing and replication speed can be inferred. Although we observed an increase in the number of fired origins, DNA replication speed was not affected by HRAS^G12V^ (Fig. S1H,I). Moreover, in whole cell lysates no abundant CHK1 phosphorylation was present, indicating inactive CHK1 and the absence of severe RS (Fig. S1J,K). However, we observed phosphorylated RPA2 in S-phase cells that overexpress oncogenic RAS, at a level comparable to mild RS induced by the topoisomerase inhibitor Aphidicolin (Fig. 1E and Fig. S1L) [37–39]. Consequently, a minor increase in γH2AX induction was shown in HRAS^G12V^ expressing cells (Fig. 1F and Fig. S1M). Moreover, we evaluated the effect of oncogenic RAS on DNA replication in living single cells, we incorporated a fluorescent tagged truncated version of 53BP1 in FUCCI4-expressing cells (Fig. 1C). The formation of 53BP1 foci is indicative of RS-induced DNA damage [5]. Using live cell imaging we saw a small but significant increase in 53BP1 foci per cell in the presence of HRAS^G12V^ in S-phase (Fig. 1G), at a level that corresponds to very mild drug-induced RS (Fig. S2A). Notably, this mild endogenous RS was specific to cells expressing oncogenic RAS and not observed upon overexpression of wild-type HRAS (Figs. S1A, S2B,C). Potentially because oncogenic RAS leads to more extreme activation of the MAPK pathway compared to overexpression of wild-type HRAS (Fig. S1B,C). To test the hypothesis that a shortened G1-phase underlies RS induced by oncogenic RAS we artificially prolonged G1-phase in our model by treating cells for 24 h with a CDK4/6 inhibitor. HRAS^G12V^ S-phase cells released from this prolonged G1-phase exhibited a partial rescue of oncogene-induced RS as shown by a reduction of 53BP1 foci (Fig. S2D,E). This shows that a shortened G1-phase in part underlies the observed RS in our model.

Although early reports showed that oncogenic RAS results in a P53-mediated growth arrest [1], we found that expression of HRAS^G12V^ in hTERT RPE-1 cells did not inhibit proliferation. This was presumably due to the presence of telomerase [40] and intact cell cycle checkpoints. However, as P53 fulfills a pivotal role in protection against RS [41, 42], we investigated if intact P53 was required to limit endogenous DNA damage in these cells. Remarkably, knockdown of *TP53* with RNAi increased the number of 53BP1 foci in HRAS^G12V^ expressing SLBP^+^/GEM^+^, S-phase cells, indicating more RS (Fig. 1H and Fig. S2F,G). Accordingly, colony formation assays with RPE cells stably expressing a short hairpin against P53 showed that P53 is required for long-term survival of oncogenic RAS expressing RPE cells (Fig. 1I and Fig. S2H–J). Collectively, these data show that acute induction of HRAS^G12V^ in unperturbed hTERT RPE-1 cells causes surprisingly mild RS, despite G1-phase shortening and unleashing malignant transformation. However, P53 is required to minimize DNA damage and maintain genomic integrity in HRAS^G12V^ expressing cells.

### Oncogenic RAS sensitizes cells to RS-inducing drugs

**Fig. 2:**
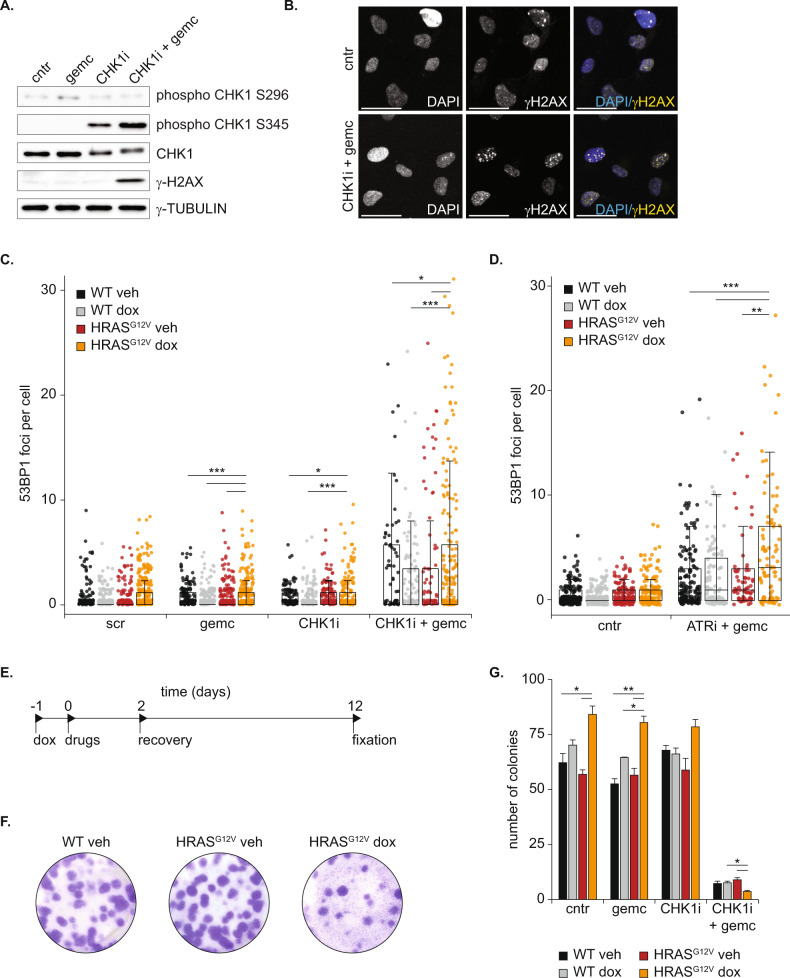
Oncogenic RAS sensitizes cells to replication stress-inducing drugs. **A** Representative immunoblot showing phosphorylation of CHK1 and γH2AX in RPE WT cells 24 h after indicated drug treatment. **B** Representative examples of γH2AX immunofluorescence staining of RPE WT cells 24 h after indicated drug treatment. Drug treatment results in a punctuated γH2AX pattern. Scale bar represents 50 μm. **C** Dot plot showing 53BP1 foci in individual RPE WT or HRAS^G12V^ cells before and 48 h after indicated drug treatment. At least 50 cells per condition were analyzed. Differences were statistically tested using a Kruskal–Wallis test with post-hoc Dunnett’s test with Benjamin Hochberg correction. **D** Same as C, but with the ATRi ceralasertib + gemcitabine. **E** Experimental setup of colony formation assay. Cells were fixed and stained 10 days after drug washout and replating. **F** Representative pictures of a colony formation assay as described in E. **G** Quantification of three colony formation assays. Bars represent mean (sum of three technical replicates, mean of three independent experiments) ± s.e.m., statistical analysis was performed with a Kruskal–Wallis test.

Excessive RS is deleterious for cell survival, and thus cancer cells are predicted to heavily rely on the intra S-phase checkpoint to control RS. Since induction of HRAS^G12V^ causes mild RS, we asked whether this would exacerbate the level of RS induced by inhibitors of the intra-S-phase checkpoint, of which ATR and CHK1 are the main players. To fully exhaust this checkpoint and induce severe RS, the ATR inhibitor Ceralasertib (ATRi) or CHK1 inhibitor Prexasertib (CHK1i) can be combined with a low dose of drugs which interfere with DNA replication such as the nucleoside analogue gemcitabine, a strategy also evaluated in clinical trials [18]. We first confirmed in normal RPE cells that 10 nM CHK1i efficiently inhibited CHK1 activation, as shown by the absence of its autophosphorylation on Serine 296 (Fig. 2A and Fig. S3A). However, only when combined with a low dose of 4 nM gemcitabine, treatment with a CHK1i resulted in RS and DNA damage, as seen by phosphorylation of CHK1 on Serine 345, a punctuated γH2AX staining, and 53BP1 foci (Fig. 2A-C and Fig. S3A,B). Notably, the low dose of gemcitabine did not induce DNA damage when applied as monotherapy indicating a synergistic effect of CHK1i + gemcitabine (Fig. 2A,C and Fig. S3A,B). The synergistic effect of CHK1i and gemcitabine was evident in both wild-type and RAS overexpressing RPE cells. Further evaluation unveiled that this combination therapy strongly slowed down DNA replication and cell proliferation independent of HRAS^G12V^ status (Fig. S3C,D). However, after 48 h of CHK1i + gemcitabine treatment the level of DNA-damage was more strongly elevated in cells overexpressing HRAS^G12V^ compared to wild-type cells, as measured by the number of 53BP1 foci and RPA2 levels per cell (Fig. 2C and Fig. S3E,F). Therefore, we focused the remainder of this study on the combination therapy of intra S-phase checkpoint inhibition and a low dose of gemcitabine.

To examine if the higher sensitivity to CHK1i + gemcitabine after induction of oncogenic RAS is drug specific, we replaced the CHK1i by an ATRi. We observed a comparable increase in 53BP1 foci (Fig. 2D), indicating that the higher sensitivity of HRAS^G12V^ cells is a general consequence of intra S-phase checkpoint inhibition. To prove that this increased sensitivity after HRAS^G12V^ induction was not cell line-specific, we repeated the experiments in U2OS osteosarcoma cells. U2OS cells are telomerase-negative, and might respond differently to oncogenic RAS than hTERT RPE-1 cells, as telomerase is shown to alleviate RS [43]. Nonetheless, U2OS cells also present mild RS upon expression of HRAS^G12V^, and were more sensitive to CHK1 i+ gemcitabine treatment (Fig. S3G–I). Because these RS-inducing drugs will not be given continuously to patients, we analyzed recovery of the cells after drug withdrawal. Colony formation assays showed that recovery from 48 h of CHK1i + gemcitabine, but not from monotherapies, was significantly impaired in RPE cells expressing oncogenic RAS compared to non-transformed RPE cells (Fig. 2E–G). In contrast, colony formation capacity under unperturbed conditions was not reduced by HRAS^G12V^ induction (Fig. 2G). Together, these data show that oncogenic RAS sensitizes cells to drug-induced RS, as seen by elevated DNA damage and impaired recovery after drug withdrawal.

### P53 signaling is essential and responsible for the response to RS

**Fig. 3:**
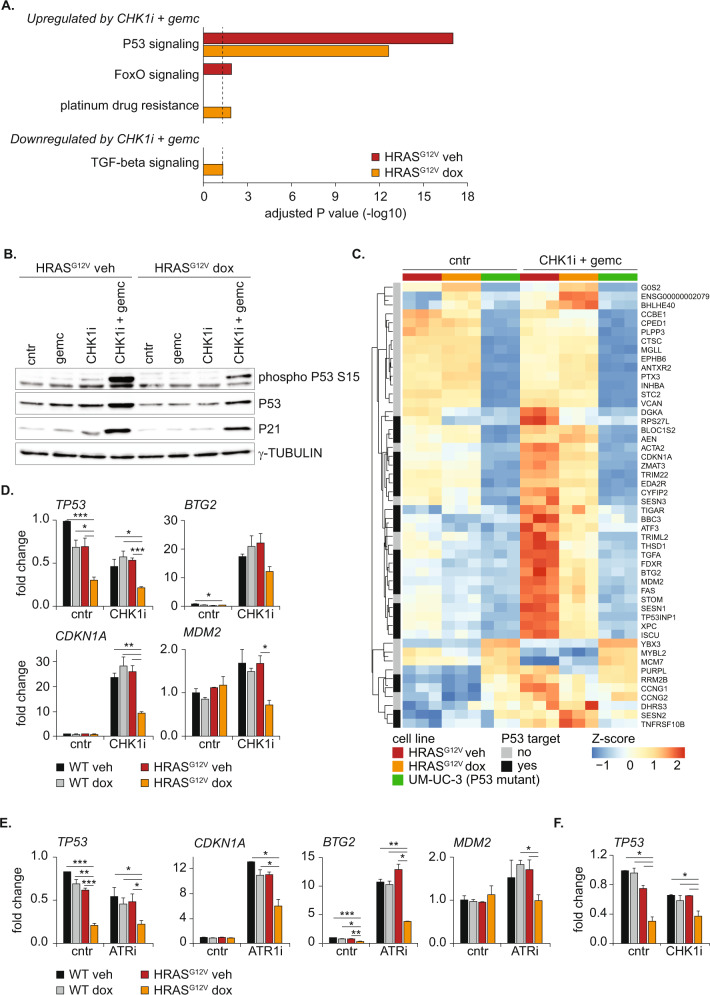
P53 signaling is essential and responsible for the response to replication stress. **A** Pathway analysis of differentially expressed genes in HRAS^G12V^ S-phase cells before and 16 h after treatment with CHK1i + gemcitabine. Significantly changed genes with a fold change of at least 1.5 were selected for analysis. This included 79 up- and 30 downregulated genes in HRAS^G12V^ veh cells and 39 up- and 43 downregulated genes in the HRAS^G12V^ dox cells. Black dotted line indicates *P* value of 0.05. **B** Representative immunoblot showing reduced P53 and P21 levels in cells with oncogenic RAS 24 h after doxycycline treatment in the absence and presence of CHK1i + gemcitabine. **C** Heatmap of the 50 most significantly changed genes in wild-type S-phase RPE cells before and 16 h after treatment with CHK1i + gemcitabine. The normalized expression of these genes in S-phase RPE cells with and without HRAS^G12V^ and UM-UC-3 S/G2-phase cells is shown. P53 target genes identified using meta-analysis are indicated [83]. **D** Quantitative PCR showing reduced expression of *TP53* and P53 target genes in HRAS^G12V^ cells before and after treatment with CHK1i + gemcitabine. Bars represent mean ± s.e.m. of 2 independent experiments. Statistical differences were evaluated using a Kruskal–Wallis test and post-hoc Dunnett’s test. **E** Same as (**D**), but now with the ATRi ceralasertib + gemcitabine. **F** Quantitative PCR of *TP53* 24 h after doxycycline administration in U2OS cells with inducible HRAS^G12V^ in the absence and presence of CHK1i + gemcitabine. Bars represent mean ± s.e.m. of two independent experiments. Statistical differences were evaluated using a Kruskal–Wallis test and post-hoc Dunnett’s test.

We set out to evaluate what underlies the accumulation of RS and impaired recovery of HRAS^G12V^ expressing cells in response to RS-inducing drugs. We hypothesized that the increased sensitivity of these cells compared to wild-type cells is caused by a different transient transcriptomic response to drug treatment. To explore gene expression programs that could explain the higher sensitivity of HRAS^G12V^-expressing cells, we performed RNA-sequencing of S-phase cells with and without HRAS^G12V^ before and 16 h after treatment with CHK1i + gemcitabine. We FACS-sorted S-phase cells using the FUCCI4 reporters to avoid bias from differences in cell cycle phase distributions. First, we evaluated the effect of overexpression of HRAS^G12V^ alone. Although induction of oncogenic RAS yielded robust transcriptional changes, they were not related to RS (Fig. S4A). We then evaluated the effect of RS-inducing drugs. In wild-type RPE cells we identified just under 100 up- and 30 downregulated genes with a fold change of at least 1.5 during drug treatment in S-phase (Fig. S4B and Table S1). Gene ontology analysis revealed that the majority of the upregulated genes is involved in P53 signaling (Fig. 3A). Accordingly, we observed stabilization and activation of P53, shown by phosphorylation at S15 and accumulation of P21, after treatment with the combination therapy of CHK1i + gemcitabine, but not with either of the two treatments alone (Fig. 3B and Fig. S4C). A subset of the P53 target genes, albeit at a lower magnitude, was also increased by CHK1i + gemcitabine treatment in HRAS^G12V^-expressing cells (Fig. 3A and Fig. S4B). Additionally, in accordance with previously published results, TGF-beta signaling was hampered in cells with oncogenic RAS (Fig. 3A) [44]. Furthermore, differential expression analysis showed that activation of the P53 program is the prime response to RS in RPE cells with and without HRAS^G12V^ overexpression (Fig. 3C).

To explore the transcriptional response to RS-inducing drugs in another cell line, we included the KRAS mutant UM-UC-3 bladder cancer cell line in our transcriptomic analysis. We chose the KRAS-mutant UM-UC-3 urothelial carcinoma cell line because it has an inactivating *TP53* mutation. This allowed us to identify RS-responses which are otherwise overwhelmed by the strong P53 activation. Furthermore urothelial carcinoma is a highly relevant cancer, because gemcitabine is part of the first line treatment regime. Exploration of a combination with intra S-phase checkpoint inhibitors thus has potential translational value. As expected, P53 target genes were not induced in the *TP53*-mutant UM-UC-3 cells after treatment with RS-inducing drugs, but remarkably no more than four other differentially expressed genes could be identified (Fig. 3C, Fig. S4B, and Table S1). The absence of alternative RS-response pathways suggests that activation of the P53 transcriptional program is the prime response to CHK1i + gemcitabine. Consistent with the lack of a P53-response, UM-UC-3 cells were highly sensitive to RS-inducing drugs, as shown by severely impaired proliferation rates and massive apoptosis upon treatment with CHK1i + gemcitabine and sensitivity to CHK1i as monotherapy (Fig. S4D–F). We did not observe cell death in hTERT RPE-1 cells, possibly due to the presence of intact cell cycle checkpoints (Fig. S4F). To evaluate the importance of P53 signaling, we treated RPE cells in which we depleted P53 using an siRNA with CHK1i + gemcitabine. This resulted in a dramatic increase in shattered nuclei, indicating mitotic catastrophes caused by cell cycle progression during drug treatment (Fig. S4G,H) [45]. Thus, our data show that transcriptional changes after intra S-phase checkpoint inhibition are P53-dependent, and underscore that P53 is essential and responsible for an efficient response to RS.

### RAS-mediated downregulation of P53 transcripts compromises the RS response in G2-phase

The data presented above underlines the importance of P53 in response to RS. Nonetheless, this P53 response upon treatment with the CHK1i + gemcitabine was dampened in cells expressing HRAS^G12V^ compared to wild-type cells (Fig. 3C). P53 activity is predominantly regulated by the stability of the P53 protein and cells with oncogenic RAS display attenuated P53 protein levels (Fig. 3B and Fig. S4C). However, since the protein translation inhibitor cycloheximide decreases P53 at similar rates in cells with and without oncogenic RAS, post-translational regulation or increased degradation rates cannot account for the lower P53 protein levels in cells with oncogenic RAS (Fig. S4I,J). Remarkably, RNA-sequencing and quantitative PCR showed that, besides prime P53 targets, also *TP53* itself was strongly downregulated in cells with oncogenic RAS under both unperturbed conditions and during CHK1i + gemcitabine treatment (Fig. 3C,D and Table S1). Similar to RS induced after treatment with a CHK1i, attenuated expression of *TP53* and its targets was observed after treatment of these cells with an ATR inhibitor (Fig. 3E). Moreover, U2OS cells with inducible HRAS^G12V^ exhibited reduced transcript levels of *TP53* (Fig. 3F). Thus, the downregulation of *TP53* by oncogenic RAS is a general response to RS-inducing drugs and is independent of cell type.

**Fig. 4:**
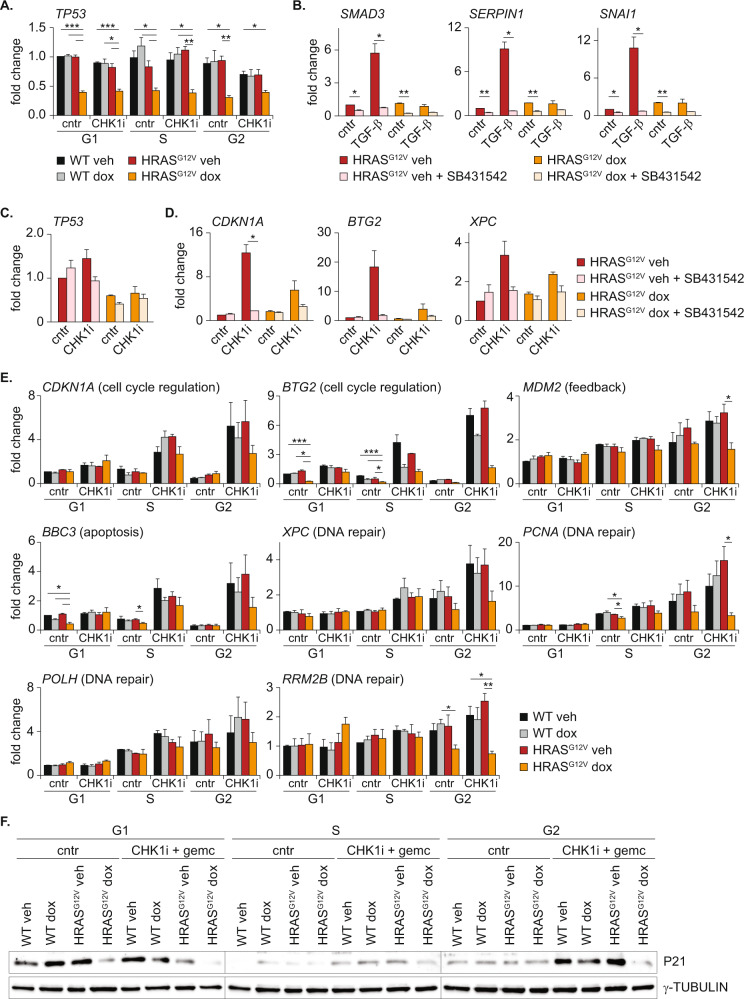
Oncogenic RAS compromises the RS response in G2-phase. **A** Quantitative PCR showing downregulation of *TP53* in HRAS^G12V^ dox cells independent of cell cycle phase. Bars represent mean ± s.e.m. of three independent experiments. Statistical differences were evaluated using a Kruskal–Wallis test and post-hoc Dunnett’s test. **B** Quantitative PCR of indicated SMAD target genes in the presence or absence of TGF-beta1 (20 ng/mL) with or without 1 μM of the TGF-beta receptor inhibitor SB431542 for 24 h. Bars represent mean ± s.e.m. of two independent experiments. Statistical differences were evaluated using a Kruskal–Wallis test and post-hoc Dunnett’s test. **C** Quantitative PCR of *TP53* before or after treatment with CHK1i + gemcitabine, with or without 1 μM of the TGF-beta inhibitor SB431542. Bars represent mean ± s.e.m. of two independent experiments. Statistical differences were evaluated using a Kruskal–Wallis test and post-hoc Dunnett’s test. **D** Quantitative PCR of indicated P53 target genes before or after treatment with CHK1i + gemcitabine, with or without 1 μM of the TGF-beta inhibitor SB431542. Bars represent mean ± s.e.m. of two independent experiments. Statistical differences were evaluated using a Kruskal–Wallis test and post-hoc Dunnett’s test. **E** Quantitative PCR of indicated P53 target genes in different cell cycle phases. Bars represent mean ± s.e.m. of three independent experiments. Statistical differences were evaluated using a Kruskal–Wallis test and post-hoc Dunnett’s test. **F** Representative immunoblot of FACS sorted RPE FUCCI4 cells showing the levels of P21 before and 16 h after treatment with CHK1i + gemcitabine in the presence or absence of oncogenic RAS.

Next, we sought to determine how oncogenic RAS signaling can transcriptionally downregulate P53. A likely candidate is the co-transcriptional regulator RAS Responsive Element Binding Protein 1 (RREB1). Oncogenic RAS phosphorylates RREB1 which cooperates with SMAD proteins to control transcription of SMAD target genes [46]. Interestingly, RREB1 is also shown to bind and activate the P53 promotor region [47], linking oncogenic RAS signaling to P53. However, knockdown of *RREB1* did not decrease *TP53* transcript levels and its downstream targets (Fig. S5A). We then exploited our RNA-sequencing dataset to identify other putative transcriptional regulators of P53 that were affected by HRAS^G12V^ induction. We selected genes with at least a 1.5-fold change in expression comparing wild-type and HRAS^G12V^ cells, DNA binding capacity and a previously described link with P53 expression (Fig. S5B). This resulted in a list of four candidates, two potential repressors (CEBP-beta and KLF4) and two potential activators (SMAD3 and KLF9) which are up- and downregulated upon HRAS^G12V^ expression respectively (Fig. S5B,C). We evaluated if siRNA oligos targeting these candidates could rescue or mimic the *TP53* levels in cells with HRAS^G12V^. Nevertheless, despite efficient depletion of CEBP-beta, KLF4 and KLF9, no rescue or phenocopy of the effect of HRAS^G12V^ on *TP53* or its targets was observed (Fig. S5D-F). However, the siRNAs targeting SMAD3 displayed conflicting results on P53 target genes (Fig. S5G–I). Therefore, we employed the TGF-beta receptor inhibitor SB431542. Although treatment with the TGF-beta receptor inhibitor completely blocked activation of classical SMAD target genes after TGF-beta1 stimulation, *TP53* levels were not affected (Fig. 4B,C). Thus, HRAS^G12V^ activation does not directly control *TP53* expression via one of the selected transcription regulators, although more elaborate studies using proteomics or screening approaches may unveil single regulators connecting oncogenic RAS to *TP53* transcription.

Besides classical activation of the P53-dependent program by P53, TGF-beta signaling is known cooperate with P53 to regulate the expression of P53 target genes [48–51]. For example, SMAD3 can bind the P21 promotor and coordinate its expression [48, 49]. We observed that oncogenic RAS downregulates transcription of TGF-beta pathway components (Fig. S1E, Fig. 3A and Fig. S4A), offering a potential additional mechanism underlying the attenuated expression of P53 target genes in HRAS^G12V^ expressing cells. First we confirmed that TGF-beta signaling is hampered in these cells. Indeed, overexpression of oncogenic RAS was sufficient to block activation of SMAD target genes after stimulation with TGF-beta1 (Fig. 4B). To test the hypothesis that attenuated TGF-beta signaling reduces the expression of P53 target genes, we inhibited TGF-beta receptor 1. This was sufficient to completely prevent transcription of P53 target genes after treatment with CHK1i + gemcitabine (Fig. 4D). Thus, oncogenic RAS attenuates the P53 response by transcriptional downregulation of P53 and TGF-beta pathway components.

The general dampened P53 response in cells with HRAS^G12V^ was identified using RNA-sequencing of S-phase cells. However, P53 regulates a plethora of genes involved in cell cycle progression, apoptosis and DNA repair whose function is not limited to S-phase. We therefore analyzed the expression of a subset of P53 target genes with different functions, before and after treatment with RS-inducing drugs and in different cell cycle phases. Although downregulation of *TP53* was evident in all cell cycle phases (Fig. 4A), most abundant downregulation of target genes was present in cells residing in G2-phase (Fig. 4E). This included genes related to DNA repair such as *PCNA* and *RRM2B*. Next we sought to evaluate if downregulation of P53 targets on a transcriptional level also results in lower protein levels. Again we FACS-sorted RPE cells, but now subjected the samples to immunoblotting. In line with the transcriptomic data, we observed reduced levels of P21 in cells expressing oncogenic RAS which was most abundant in G2 phase (Fig. 4F and Fig. S6A). In a second approach to test protein levels of P53 target genes, we performed immunofluorescence staining of RPE cells harboring the FUCCI4 cell cycle markers. This revealed downregulation of RRM2B protein in S and G2-phase in cells expressing HRAS^G12V^ compared to wild-type cells after treatment with CHK1i + gemcitabine (Fig. S6B,C). Furthermore, P21 protein levels were dampened irrespective of cell cycle stage (Fig. S6D,E). These data show that oncogenic RAS blocks the expression of P53 and its target genes during the RS response, resulting in strong downregulation of multiple P53-responsive DNA repair genes and cell cycle inhibitors.

### HRAS^G12V^ exacerbates level of RS and delays cell cycle exit during drug treatment

**Fig. 5:**
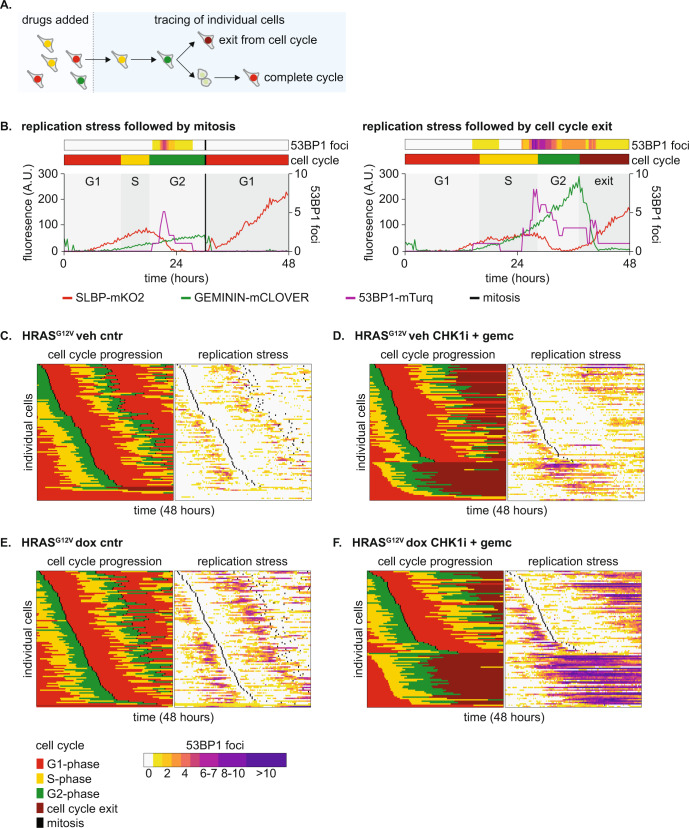
Single cell analysis of the replication stress response. **A** Schematic representation of cell tracing strategy and potential fates in live cell imaging experiments. **B** Representative cell traces of two cells showing 53BP1 foci and cell cycle progression in the presence of CHK1i + gemcitabine. The left cell completes mitosis, the right cell exits the cell cycle without mitosis. **C** Heatmap showing cell cycle progression and 53BP1 foci in 100 individual HRAS^G12V^ veh cells under control conditions. Each row represents a single cell. Cells were ordered based on the moment of first mitotic entry. **D** Same as C, but now cells were treated with CHK1i + gemcitabine. **E** Same as C, but now HRAS^G12V^ dox cells. **F** Same as C, but now HRAS^G12V^ dox cells that were treated with CHK1i + gemcitabine.

**Fig. 6:**
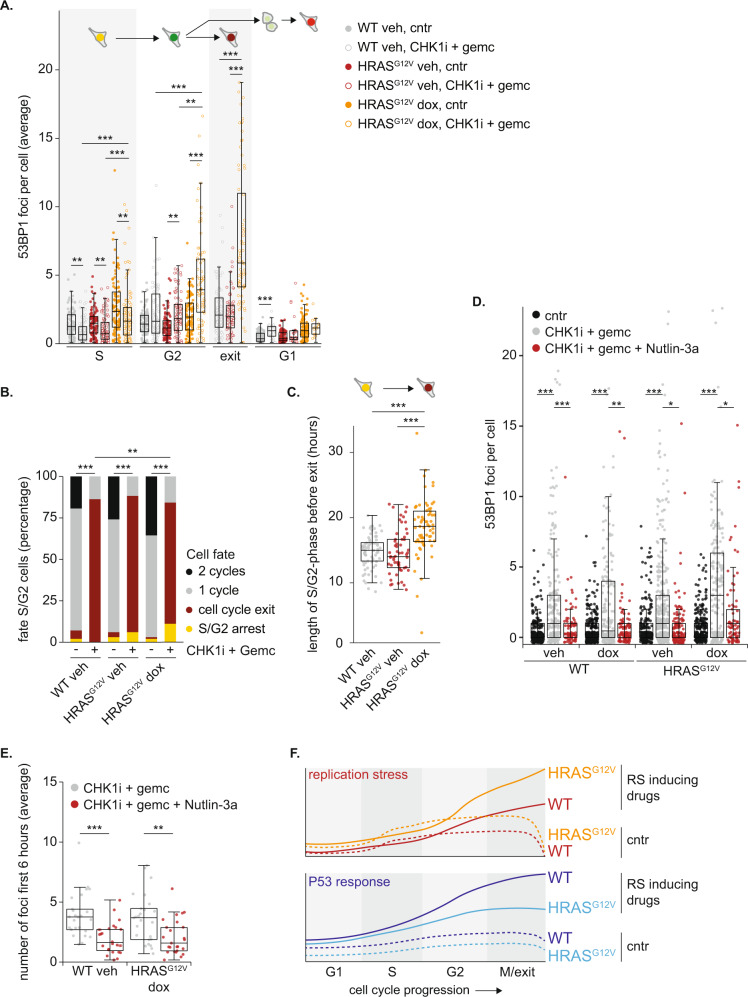
P53 silencing by HRAS^G12V^ exacerbates RS during drug treatment. **A** Quantification of live cell imaging data showing the average number of 53BP1 foci per image per cell in different cell cycle phases. Cells were included from S-phase entry onwards. 100 cells were analyzed, and statistical analysis was performed with a Kruskal–Wallis test with post-hoc Dunnett’s test with Benjamin Hochberg correction. **B** Bar chart showing the frequencies of different cell fates of S-phase cells after treatment with CHK1i + gemcitabine. 100 cells per condition were evaluated and statistical difference was tested with a Chi-square test corrected for multiple comparisons. **C** Dot plot in which the duration from S-phase entry until cell cycle exit is shown. Only cells which enter S-phase and subsequently exit the cell cycle upon treatment with CHK1i + gemcitabine were included. Statistical differences were evaluated using a Kruskal–Wallis test. **D** Quantification of 53BP1 foci per cell, cultured for 48 h in the presence or absence of CHK1i + gemcitabine combined with Nutlin-3a. Statistical differences were evaluated using a Kruskal–Wallis test. **E** Quantification of the live cell imaging data shown in Fig. S7D–E. Average number of 53BP1 foci in S/G2-phase cells during the first 6 h of treatment were plotted. 25 cells per condition were analyzed. Statistical differences were evaluated using a Kruskal–Wallis test and post-hoc Dunnett’s test. **F** Schematic overview representing the key findings of the current study. Oncogenic RAS induces endogenous RS which can be exacerbated by treatment with RS-inducing drugs. Activation of the P53 transcription program is the prime response to RS. This response is compromised in cells with HRAS^G12V^ due to transcriptional downregulation of P53.

Since P53 is a critical regulator of cell cycle progression and DNA repair, the dampened P53 response during S/G2-phase in cells with oncogenic RAS, can be expected to have profound impact on cell cycle fates. To evaluate this at a single cell level, we performed a live cell imaging experiment. We treated cells expressing the FUCCI4 markers as well as truncated 53BP1, to monitor DNA damage, with a CHK1i + gemcitabine and followed the fates of individual cells in response to treatment (Fig. 5A,B). To evaluate cell fates, single cell traces (Fig. 5B) were combined in heatmaps, and ordered according to the moment of the first mitosis (Fig. 5C–F). These heatmaps confirmed that cells with HRAS^G12V^ experience elevated levels of endogenous RS, as measured by 53BP1 foci during S/G2-phase, which was exacerbated upon treatment with RS-inducing drugs (Fig. 5C–F and Fig. 6A). Although CHK1i + gemcitabine treatment initially decreased the average number of 53BP1 foci throughout S-phase in all cell lines, elevated levels of RS-induced DNA damage in cells with oncogenic RAS were clearly evident during G2-phase (Fig. 6A). Offspring of cells that were able to undergo mitosis showed a low number of 53BP1 foci in the next G1 (Fig. 6A). These foci in G1 cells, usually referred to as nuclear bodies, indicate unresolved DNA damage, but did not differ substantially between any of the conditions [37].

Although some cells completed mitosis in the presence of RS-inducing drugs, the majority exited the cell cycle by premature activation of APC/C^CDH1^, as seen by the loss of Geminin without mitosis (Fig. 5B-F and Fig. 6B). We asked if cells which exit the cell cycle in this manner experienced the highest levels of RS. However, when we classified cells according to cell cycle fate and analyzed the number of foci in the initial S-phase, we observed that cell cycle exit was not necessarily preceded by an S-phase with the highest levels of RS-induced DNA damage (Fig. S7A). Instead, 53BP1 foci accumulate after exit from the cell cycle, specifically in cells with oncogenic RAS (Fig. 5C-F and Fig. 6A). This marked increase in 53BP1 foci in HRAS^G12V^ expressing cells exiting the cell cycle most likely represents a failure to repair DNA breaks [52].

Despite elevated levels of RS-induced DNA damage in G2-phase cells with oncogenic RAS compared to wild-type cells, the cell cycle exit in CHK1i + gemcitabine treated cells after completion of S-phase was delayed by HRAS^G12V^ expression (Fig. 6B,C). Potentially, the higher level of DNA damage in these cells has slowed down S-phase in a graded fashion [53], which could subsequently delay cell cycle exit. Additionally, P53-dependent P21 accumulation plays an important role in re-activating APC/C^CDH1^ to enforce the cell cycle exit [54]. This P21 accumulation was impaired in cells expressing HRAS^G12V^ (Fig. 4E,F and Fig. S6D,E). Thus, oncogenic RAS exacerbates the level of RS-induced DNA damage upon intra S-phase checkpoint inhibition and delays exit from the cell cycle.

HRAS^G12V^ transcriptionally downregulates P53 resulting in attenuated activation of target genes which play a pivotal role in DNA repair. To bear out the hypothesis that a dampened, P53-induced, DNA-repair response directly underlies the increase in RS observed in cells with oncogenic RAS, we stabilized P53 using the MDM2 inhibitor Nutlin-3a. Nutlin-3a stabilized P53 target genes, but could not rescue the mild endogenous RS in cells with oncogenic RAS (Fig. S7B,C). In contrast, Nutlin-3a resulted in marked decrease in 53BP1 foci formation after 48 h of treatment with CHK1i + gemcitabine (Fig. 6D). Sustained P53 signaling is proven to affect cell fate and enforce a cell cycle arrest [55]. Indeed, live cell imaging revealed that Nutlin-3a forced the vast majority of cells which were in early S-phase upon treatment with RS-inducing drugs to exit the cell cycle (Fig. S7D,E). Despite this cell cycle exit, we observed that Nutlin-3a rescued the formation of 53BP1 foci, indicating less RS, during the first hours after the start of drug treatment (Fig. 6E). Prolonged Nutlin-3a incubation in S-phase cells did not prevent the formation of 53BP1 foci (Fig. S7D,E). Most likely, the continuous Nutlin-3a-induced accumulation of P53 leads to extremely high levels of its target P21 that completely blocks CDK activity and thereby impedes DNA replication and G2-phase progression [56].

Collectively, these data demonstrate that intra S-phase checkpoint inhibition exacerbates RS in cells with oncogenic RAS via transcriptional downregulation of P53 and TGF-beta pathway components. Thus, our data predict that RAS mutations enhance sensitivity and impair recovery of cancer cells to treatment with RS-inducing drugs.

## Discussion

Oncogenes are described to confer sensitivity to ATR and CHK1 inhibition due to the presence of endogenous RS [13–15, 57]. We show that oncogenic RAS prompts expedited S-phase entry, which is previously shown to result in firing of intragenic origins [35]. Since both DNA replication and transcription use DNA as a substrate, co-occurrence of replication and transcription leads to collisions between the two machineries and elevated levels of endogenous RS. In addition, oncogenic RAS augments global transcription rates [26], further increasing the risk of collisions. Accordingly, RPE HRAS^G12V^ cells display mild endogenous RS, which is exacerbated upon treatment with inhibitors of the intra S-phase checkpoint. Since depletion of members of the transcription complex prevented collisions between replication and transcription machineries and alleviated RS induced by ATR inhibitors [58], it can be suggested that oncogene-induced transcription-replication conflicts are a main cause of sensitivity to CHK1i + gemcitabine treatment.

However, the mechanistic experiments presented here reveal that, besides endogenous RS, P53 is a major determinant in sensitivity to RS-inducing drugs. Furthermore, we found that oncogenic RAS dampens P53 signaling which sensitizes cells to RS. Although frequently mutated, P53 is intact in a substantial percentage of tumors harboring mutated RAS [59]. The mutation status of P53 has been proposed to be predictive for sensitivity to ATR and CHK1 inhibitors, but clinical trials have not confirmed this yet [21]. Thus, we propose that mechanisms controlling P53 activity such as RAS-dependent signaling must be taken into account when predicting sensitivity to intra-S-phase checkpoint inhibitors in individual cancer patients. In line with this, our study provides a rationale to select cancer patients with hyperactivation of the MAPK pathway for treatment with intra S-phase checkpoint inhibitors. In contrast, therapies combining inhibitors of MAPK signaling and RS-inducing drugs should be carefully evaluated.

We detected attenuated P53 transcript levels in HRAS^G12V^ cells irrespective of cell cycle phase. Nonetheless, downregulation of target genes was mainly observed in S/G2-phase. Hence, it can be proposed that P53 executes its protective function during DNA replication, rather than by preventing cell proliferation [60]. Indeed, prime P53 target genes involved in cell cycle arrest and apoptosis are dispensable for tumor suppression in vivo [61]. This leaves energy metabolism, DNA repair, and preservation of genomic integrity as the key tumor-suppressive functions of P53. In line with a role in protecting DNA from damage, P53 is shown to prevent slowing and stalling of replication forks under conditions of genotoxic stress [42]. This effect was at least in part dependent on the P53 target *MDM2*, which we also found downregulated in cells overexpressing oncogenic RAS (Fig. 4B). In addition, we observed that the P53 target *POLH*, which encodes the translesion synthesis (TLS) polymerase η, was downregulated by HRAS^G12V^. TLS polymerases are required to facilitate DNA elongation over damaged DNA. Although this increases the risk of mutagenesis, it enhances DNA damage tolerance [62]. Recent work demonstrated that polymerase η is indeed essential for recovery from RS-inducing drugs [63]. In addition to the canonical, transcriptional-dependent, function of P53, a growing body of evidence indicates that P53 also executes its function directly at the DNA replication fork [64, 65], all together pointing towards a prime function for P53 during S/G2-phase of the cell cycle.

Our live cell imaging data builds upon previous publications which show that cells exit the cell cycle when they encounter severe levels of RS [54, 66]. It is conceivable that such an exit is important to maintain genomic integrity. Notably, this RS-induced cell cycle exit was delayed in cells with oncogenic RAS (Fig. 6C). Several mechanisms can potentially attribute to a prolonged S/G2-phase. Firstly, the speed of S-phase progression correlates with the level of DNA damage. Since HRAS^G12V^-expressing cells experience more RS than wild-type cells during treatment with CHK1i + gemcitabine, this might result in a more profound stalling of DNA replication and subsequently delay the cell cycle exit [53]. However, our fiber assays did not show difference in fork speed between cells with and without oncogenic RAS, suggesting that another putative mechanism is in place. This second mechanism involves the P53-P21 pathway, which is at least in part responsible for cell cycle exit by inhibition of CDK2 [66–68] and which absence delays cell cycle exit [54]. In line with this, it can be hypothesized that the reduced levels of P21 observed in HRAS^G12V^ G2-phase cells contribute to a delayed cell cycle exit from G2-phase.

We have previously shown that cells which exit the cell cycle in G2-phase are not permanently arrested but can re-enter the cell cycle [66]. Also after severe drug-induced RS recovery of HRAS^G12V^-transformed cells occurs, albeit at a lower level (Fig. 2E-G). In light of cancer therapy it is of great importance to understand how oncogenic RAS can interfere with this arrest. Reyes and coworkers demonstrated that rare cells which escape a DNA damage-induced cell cycle arrest are characterized by a lower P53 pulse amplitude [69]. In such a scenario oncogenic RAS would facilitate cell cycle re-entry by lowering P53 levels. In support of this notion, it was determined that intermediate levels of P21 create a sweet spot for escape from senescence [70]. The decision to proliferate or arrest after DNA damage is determined by competing actions of the P53-P21 axis and ERK activity. The inhibitory effect of oncogenic RAS on TP53 and TGF-beta pathway components dampens P21 levels, whereas ERK promotes cell cycle entry by stimulating Cyclin D-CDK activity [30]. This combined action of oncogenic RAS signaling could tip the balance to cell proliferation at the expense of increased genomic instability. Thus, although RAS-mutant cells accumulate more DNA damage after replication stress, rare cells might be able to escape a G2 arrest. This increases the chance of mutations which can confer drug resistance resulting in tumor relapse.

Besides the pivotal role of P53 in anti-cancer therapy responses, the finding that oncogenic RAS downregulates P53 sheds a new light on tumor evolution. The lack of P53 fulfills a central function in tumorigenesis by evading growth suppressors and resisting programmed cell death [71]. This induces genomic instability which is essential to acquire oncogenic traits. Despite the key role of P53 in tumorigenesis, it is mutated relatively late in the process of malignant transformation [72, 73]. On this basis, it is tempting to speculate that alternative mechanisms are required to loosen the stringency of the P53-induced checkpoint early during tumor development. In line with this, it is shown that the transcriptional output of wild-type P53 correlates with breast cancer tumorigenesis [74]. Moreover, reduced levels of P53 could directly contribute to genomic instability as heterozygous deletion of P53 induces RS [42]. In such a scenario, the joined induction of oncogenic RS and P53-checkpoint dampening by activation of oncogenes, such as RAS, would form the basis for malignant transformation.

## Materials and methods

### Key resources

Key resources are listed in Table S2.

### Cell lines and cell line generation

hTERT RPE-1, HEK293T, U2OS and UM-UC-3 cell lines were purchased from ATCC. The hTERT-RPE1 cells stably expressing the short hairpin against P53 #1 as described in [75] were a kind gift from Prof. Rene Bernards. hTERT RPE-1, U2OS and HEK293T cells were cultured in DMEM, UM-UC-3 cells were cultured in EMEM. All media were supplemented with 10% FBS and 1% pen/strep and all cells were cultured at 37°C, 5% CO_2_. All cell lines were regularly tested and confirmed mycoplasma negative.

Gemcitabine, Prexasertib, Palbociclib, Ceralasertib and SB431542 were purchased from Selleck chemicals, Nutlin-3a and cycloheximide were purchased from Sigma and used at a final concentration of 4 nM, 10 nM, 1μM, 1μM, 1μM, 1μM and 100μg/mL respectively, unless stated otherwise. TGF-beta 1 was purchased from Immunotools and used at a final concentration of 20 ng/mL.

RPE cell lines harboring the Tet Repressor, FUCCI4 system, fluorescently tagged H2B, 53BP1 and HRAS^G12V^ were created using lentiviral transduction with the third-generation lentiviral packaging system as previously described [76]. In brief, HEK293T cells were transfected with 10 μg lentiviral packaging plasmids and 10 μg of the construct of interest using PEI. After 2 h transfection, medium was washed away, lentivirus containing medium was harvested after 48 h. RPE cells were transduced with lentivirus containing medium supplemented with 8μg/mL Polybrene for 24 h.

The lentiviral constructs encoding mKO2-SLBP(18-126) and Clover-Geminin(1-110) were a gift from Michael Lin (Addgene plasmid # 83915; RRID:Addgene_83915, Addgene plasmid # 83914; RRID:Addgene_83914). The plasmid encoding Apple-53BP1trunc was a gift from Ralph Weissleder (Addgene plasmid # 69531; RRID:Addgene_69531). The plasmid encoding pLenti-H2B-iRFP720 was a gift from Carlos Carmona-Fontaine (Addgene plasmid # 128961; RRID:Addgene_128961). The plasmid encoding HRAS^G12V^ was a gift from Judith Campisi. The plasmid encoding wild-type HRAS was created by reverting the G12V mutation back to the wild-type sequence using the Q5 Site-Directed Mutagenesis kit according to the manufacturers’ instructions (NEB, E0555S). The fluorescent tag of truncated 53BP1 was changed to mTurquoise2 using Gibson Assembly. Cells harboring the Tet repressor or HRAS were selected using blasticidin (10μg/ml, 10 days) and puromycin (1.0μg/ml, 5 days) respectively. Cells harboring fluorescent tagged SLBP, Geminin, 53BP1 and H2B were selected by FACS-sorting.

### Mouse experiments

Animal experiment were approved by the Utrecht University Animal Ethics Committee (approval no. AVD108002016626) and performed according to institutional and national guidelines. For xenograft experiments, 1 million cells in 200 μL basic DMEM were injected in the lower and upper flanks of immunocompromised Rj:NMRI-Foxn1nu/nu mice (Janvier Labs), a total of two mice was used. No randomization of samples was used. Doxycycline (2 g/kg) was administrated ad libitum in pellets to all injected mice (Bio Services). Tumor size was monitored biweekly, and mice were euthanized when one tumor exceeded the size of one cubic centimeter. No samples were excluded from analysis. Tumors were harvested and stored in paraformaldehyde.

### RNAi transfections

For siRNA experiments, cells were transfected with a final concentration of 10 nm siRNA targeting the gene of interest or a scrambled control using Lipofectamine RNAiMAX according to manufacturers’ instructions (Life Technologies, 13778030). The following siRNAs were used: Dharmacon D‐001210‐02‐05 (Scrambled), LQ-003329-00-0002 (TP53), LQ-019150-00-0002 (RREB1), LQ-006423-00-0005 (CEBPβ), LQ-005089-00-0002 (KLF4), LQ-020067-00-0002 (SMAD3) and LQ-011223-00-0002 (KLF9).

### Microscopy

Live cell imaging experiments and downstream analysis were performed as previously described [66]. In brief, 1500 RPE cells were seeded in a CELLview slide (Greiner). Images were acquired at 20-minute time intervals for 48 h in a humidified chamber on a Nikon A1R-STORM microscope using a 40x LWD objective. Cell tracking and foci analysis was performed in a semi-automated manner using the FIJI plugin Trackmate.

For RPE cells, images for 53BP1 foci analysis of snapshots were acquired on a NIKON Ti-E microscope and foci quantification was performed manually using FIJI.

For immunofluorescence staining, cells were seeded on coverslips, fixed using 4% paraformaldehyde for 20 min and permeabilized with 0.1% Triton X-100 for 1 min at room temperature. Samples were blocked with 5% goat serum and incubated with antibodies prior to mounting on slides. Slides were analyzed on a Leica SP8 confocal microscope using a 20x objective. For analysis, ROIs were determined based on the DAPI signal. For RPA2 and γH2AX staining, 1 min pre-extraction with 0.2% Triton X-100 prior to paraformaldehyde fixation was performed. Foci per cell were determined with a custom-made FIJI script using the Find Maxima function. 53BP1 foci in U2OS cells were quantified manually. For each condition at least 100 cells were quantified. Antibodies used and dilutions are listed in Table S3.

DNA fiber assays were performed as described previously [76]. Briefly, cells were pulsed with CldU (25 μM) for 20 min, washed with PBS and pulsed with IdU (250 μM) for 20 min. Cells were collected, lysed in spreading buffer (200 mM Tris-HCl pH 7.4, 50 mM EDTA, 0.5% SDS), and spread on a microscope slide under an angle of 15 degrees. Cells were fixed in methanol-acetic acid (3:1) and treated with 2.5 mM HCl for 75 min prior to blocking and antibody incubation. Slides were analyzed on a Leica SP8 confocal microscope with 63x objective. Length and type of DNA fibers were analyzed in FIJI using the length measurement tool.

### Flow cytometry

For cell sorting experiments, cells were resuspended in DMEM and filtered with 40 μm cell strainers to remove cell clumps. Cell sorting was performed on a BD FACS Fusion flow cytometer based on fluorescent intensity for mKO2-SLBP and mClover-Geminin. For each condition 100,000 cells were sorted and collected in ice-cold PBS.

For Annexin V staining experiments, 100,000 cells were collected by trypsinization, washed with PBS and stained with Annexin V-Alexa 647 antibodies according to manufacturers’ instructions (Thermo Fisher, A23204). Next samples were loaded on a CytoFLEX flow cytometer. Quantification of Annexin V positive cells was conducted using FlowJo v10.0 software.

For γH2AX and phospho RPA2-S8 staining, cells were fixed using 4% PFA for 30 min and permeabilized using 0.1% Triton. Cells were washed with 0.1% BSA in PBS prior to incubation with antibodies for 1 h at room temperature. DAPI was added to stain DNA content. Samples were loaded on a CytoFLEX flow cytometer and analyzed using FlowJo v10.0 software.

### Immunoblotting

For immunoblotting cells were washed with ice-cold PBS and lysed in ice-cold RIPA-buffer (50 nM Tris-HCl pH 7.5, 1 mM EDTA, 150 mM NaCl, 0.25% deoxycholate, 1% NP-40) supplemented with NaF (1 mM), NaV_3_O_4_ (1 mM) and protease inhibitor cocktail (11873580001, Sigma Aldrich) after which samples were subjected to a standard SDS-page immunoblot. Antibodies used and dilutions are listed in Table S3.

### RNA-sequencing

For RNA-sequencing S-phase (Geminin-positive/SLBP-positive) RPE and S/G2-phase (Geminin-positive) UM-UC-3 cells were collected using FACS sorting. For each sample three biological replicates were used, and two independent clones of RPE cells harboring the FUCCI4 reporter system were included. RNA was isolated using the QIAGEN RNAeasy kit according to manufacturers’ recommendations. Next, samples were subjected to sequencing and single-end reads were checked for quality, aligned, and counted using the USEQ RNA-seq pipeline (version 2.3.0). Briefly, a quality check with FastQC (version 0.11.4) was performed [77], reads were aligned to the human genome (hg19) with STAR (version 2.4.2a) [78] after which post alignment processing and quality control was done with sambamba (version 0.5.8) [79], Picard-toolkits (version 1.141) and bamMetrics (version 2.1.4) respectively. Final read counts were obtained using HTSeq-count (version 0.6.1) [80]. Raw read counts were further analysed with DEseq2 (version 1.28.0) [81] using default analysis parameters and the differential gene expression between groups was assessed as shrunken log2 fold changes (LFC). Differential expression analysis was performed using R (version 3.6.3) and RStudio Desktop (version 1.3.1093). Pathway analysis was performed on significantly changed genes with a fold change of at least 1.5 (Table S1) with the ToppGene application using KEGG pathways [82]. Sequencing data is available on Gene Expression Omnibus under accession number GSE168987.

### Quantitative PCR

RNA isolation (QIAGEN, RNAeasy kit), synthesis of cDNA (Thermo Fisher) and qPCR (Bio-Rad, SYBR green master mix) were performed according to manufacturers’ guidelines. GAPDH and 18 S were used as reference genes. Fold changes were calculated using the ΔΔCt method. Primers used in this manuscript are listed in Table S4.

### Colony formation assay

For colony formation assays cells were plated at low density (250 cells/well) in a 6 well plate. Medium was replenished every 48 h and cells were fixed after 10 days. Fixation was performed with Acitic acid:Methanol (1:7 vol/vol) for 5 min, then cells were washed with PBS and incubated with Crystal Violet staining solution (0.5% Crystal Violet, Sigma-Aldrich) for 2 h at room temperature. Subsequently, dishes were rinsed with water and air-dried. Number of colonies per well was counted manually, each condition was performed in triplicate.

### Quantification and statistical analysis

Microscopy, immunoblot and quantitative PCR experiments were performed three times unless indicated otherwise. Please note that sometimes values appear below zero, for example in Fig. 1H, this is due to vertical jitter. Immunoblots shown are representative examples of at least two independent experiments. Quantification of immunoblots was performed using the rectangular selection tool in ImageJ. Briefly, band density of protein of interest was measured, corrected for loading by the γ-tubulin signal after which all samples were normalized to the control sample. Statistical analysis performed as indicated in figure legends. Requirement to perform statistical tests were met. Statistical analysis of qPCR experiments and 53BP1 foci per cell was done by a Kruskal–Wallis test with a post-hoc Dunnett’s test with Benjamin Hochberg correction. *P < 0.05, **P < 0.01, ***P < 0.001.

## Supplementary information

Supplemental table 1

supplemental material

## Data Availability

The custom made scripts used for data analysis in this manuscripts are available upon request.
