# Supplementary material for: Oncogenic RAS sensitizes cells to drug-induced replication stress via transcriptional silencing of P53
Source: Oncogene. 2022 Apr 7;41(19):2719–33. doi: 10.1038/s41388-022-02291-0 (PMC9076537; doi:10.1038/s41388-022-02291-0)
Supplement: Supplementary file 2 — supplemental material [file 41388_2022_2291_MOESM2_ESM.pdf]

Supplementary information

Oncogenic RAS sensitizes cells to drug-induced replication stress via transcriptional silencing of P53

Hendrika A. Segeren, Elsbeth A. van Liere, Frank M. Riemers, Alain de Bruin, Bart Westendorp

1. Supplementary Tables

Table S1. RNA-sequencing of FACS-sorted S-phase cells

Table S1 is available in the online version of this manuscript.

Table S2. Key resources

Reagent or resource	Source	Identifier
**Antibodies**		
Anti-53BP1	Novus Biologicals	NB100-304, RRID:AB_10003037
Rabbit monoclonal anti CHK1, Phospho (Ser345)	Cell Signaling Technology	2348; RRID:AB_331212
Rabbit monoclonal anti CHK1, Phospho (Ser296)	Cell Signaling Technology	2349; RRID:AB_2080323
Mouse monoclonal anti-CHK1	Cell Signaling Technology	2360; RRID:AB_2080320
Rabbit monoclonal anti-ERK1/2, phospho (T202/204)	Cell Signaling Technology	4370; RRID:AB_2315112
Rabbit polyclonal anti-ERK1	Santa Cruz Biotechnology	sc-94; RRID:AB_2140110
Rabbit monoclonal anti-SMAD3	Cell Signaling Technology	9523; RRID:AB_2193182
Rabbit polyclonal anti-P21	Santa Cruz Biotechnology	sc-471; RRID:AB_632123
Mouse monoclonal anti-P53	Santa Cruz Biotechnology	sc-126; RRID:AB_628082
Rabbit monoclonal anti-p53R2 (RRM2B)	Thermo Fisher	MA5-29529; RRID:AB_2785378
Mouse monoclonal anti-gamma-Tubulin	Sigma-Aldrich	T6557; RRID:AB_477584
Rabbit anti-Phospho-Histone H2A.X (Ser139)	Cell Signaling Technology	2577; RRID:AB_2118010
Alexa Fluor 647 anti-H2A.X phospho (Ser139)	BioLegend	613408; RRID:AB_2295046
Rabbit anti-RPA2 phospho S8	Cell Signaling Technology	54762; RRID:AB_2799471
Rat anti-BrdU	Bio-Rad	OBT0030G RRID:AB_609567
Mouse anti-BrdU	BD Biosciences	347580 RRID:AB_10015219
**Chemicals**		
Palbociclib	Selleck chemicals	S1116
Gemcitabine	Selleck chemicals	S1714
Prexasertib	Selleck chemicals	S7178
Ceralasertib	Selleck chemicals	S7693
SB431542	Selleck chemicals	S1067

Nutlin-3a	Sigma-Aldrich	SML0580
DAPI	Sigma-Aldrich	D9542
Protease inhibitor Cocktail	Sigma-Aldrich	11873580001
Fetal bovine serum	Thermo Fisher	10500064
DMEM	Thermo Fisher	41966052
EMEM	LGC	30-2003
**Critical commercial assays**		
Rneasy Mini Kit for RNA extraction	Qiagen	74106
Rneasy Micro Kit for RNA extraction	Qiagen	74004
Annexin V, Alexa Fluor™ 647 conjugate	Thermo Fisher	A23204
Q5 Site-Directed Mutagenesis kit	New England Biolabs	E0554S
**Deposited data**		
RNA sequencing RPE-FUCCI4 HRAS^{G12V} and UM-UC-3 cells	This paper	GEO: GSE168987
**Experimental Models**		
hTERT RPE-1	ATCC	CRL-4000; RRID:CVCL_4388
hTERT RPE-1 shP53	Gift from Rene Bernards lab	N/A
HEK293T	ATCC	CRL-3216; RRID:CVCL_0063
UM-UC-3	ATCC	CRL-1749; RRID:CVCL_1783
U2OS	ATCC	HTB-96; RRID:CVCL_0042
mice	Janvier Labs	Rj:NMRI-Foxn1nu/nu mice
**Oligonucleotides**		
Primers used for qPCR, see Table S1	This paper	N/A
Scrambled siRNA	Dharmacon	D-001210-02-05
Human P53 siRNA	Dharmacon	LQ-003329-00-0002
Human RREB1 siRNA	Dharmacon	LQ-019150-00-0002
Human CEBPB siRNA	Dharmacon	LQ-006423-00-0005
Human SMAD3 siRNA	Dharmacon	LQ-020067-00-0002
Human KLF4 siRNA	Dharmacon	LQ-005089-00-0002
Human KLF9 siRNA	Dharmacon	LQ-011223-00-0002
**Recombinant DNA**		
pLenti CMV TetR Blast (716-1)	Addgene	Addgene_17492
pLenti CMV HRAS^{G12V} Puro	Gift from Judith Campisi	N/A
Apple-53BP1trunc	Addgene	Addgene_69531
Clover-Geminin(1-110)	Addgene	Addgene_83915

mKO2-SLBP(18-126)	Addgene	Addgene_83914
H2B-iRFP670	Addgene	Addgene_128961
pMDLg/pRRE lentiviral packaging	Addgene	Addgene_12251
pRSV-Rev lentiviral packaging	Addgene	Addgene_12253
pCMV-VSV-G lentiviral packaging	Addgene	Addgene_8454
**Software and Algorithms**		
FIJI (ImageJ)	https://fiji.sc	RRID:SCR_002285
TrackMate	https://imagej.net/TrackMate	N/A
NIS elements	Nikon	RRID:SCR_014329
FlowJo	BD	RRID:SCR_008520
R	https://www.R-project.org/	N/A
Rstudio	https://www.rstudio.com/	RRID:SCR_000432
USEQ RNA-seq pipeline	https://github.com/UMCUGenetics/RNASeq	N/A

Table S3. Antibodies for immunoblots and immunofluorescence staining

Application	***Name***	***Company***	***Catalogue number***	***Dilution***
Immunoblots	CHK1 phospho S296	Cell Signaling	2349	1:1000
	CHK1 phospho S345	Cell Signaling	2348	1:1000
	CHK1	Cell Signaling	2360	1:1000
	ERK1/2 phospho T202/204	Cell Signaling	4370	1:1000
	ERK1	Santa Cruz	sc-94	1:1000
	γ-H2AX (S139)	Cell Signaling	2577	1:1000
	P21	Santa Cruz	sc-471 (M-19)	1:1000
	P53	Santa Cruz	sc-126	1:1000
	SMAD3	Cell Signaling	9523	1:1000
	γ-Tubulin	Sigma Aldrich	T6557 (GTU-88)	1:1000
Immunofluorescence	53BP1	Novus Biologicals	NB100-304	1:2000
	γ-H2AX (S139)	Cell Signaling	2577	1:200
	P21	Santa Cruz	sc-471 (M-19)	1:800
	RPA2	Millipore	MABE285	1:500
	RRM2B	Thermo Fisher	MA5-29529	1:100
	Rat anti-BrdU	Bio-Rad	OBT0030G	1:100
	Mouse anti-BrdU	BD Biosciences	347580	1:100
Flow cytometry	Alexa Fluor 647 anti-H2AX phospho (Ser139)	BioLegend	613408	1:200
	RPA2 phospho S8	Cell Signaling	54762	1:500

Table S4. qPCR primers

Gene	***Forward primer (5'-3')***	***Reverse primer (3'-5')***
BTG2	GGCTTAAGGTCTTCAGCGGG	TGTGGTTGATGCGAATGCAG
BBC3	GAGCAGGGCAGGAAGTAACA	CACAAATCTGGCAGGGGACC
CDKN1A	CTCTAAGGTTGGGCAGGGTGACC	CAGAGGGGGGTATCAAGAGCCAG
CDKN1B	AATGCCGGTTCTGTGGAGC	ATGTCCATTCCATGAAGTCAGC
CEBPβ	TTTGTCCAAACCAACCGCAC	GCATCAACTTCGAAACCGGC
CCND1	GTCCATGCGGAAGATCGTC	AGGCCACGAACATGCAAGTG
GAPDH	CTCTGCTCCTCCTGTTCG	GCCCAATACGACCAAATCC
HRAS	GGAAGCAGGTGGTCATTGAT	ATGGCAAACACACACAGGAA
KLF4	CATCTCAAGGCACACCTGCGAA	TCGGTCGCATTTTTGGCACTGG
KLF9	GCCGCCTACATGGACTTCG	GGATGGGTCGGTACTTGTTCA
MDM2	CGAGCTTGGCTGCTTCTGG	GTACGCACTAATCCGGGGAG
PCNA	GGTTACTGAGGGCGAGAAGC	GCTGAGACTTGCGTAAGGGA
POLH	ACTGGCACAAGTTCGTGAGT	CATCAATGCTGGCACGTTCA
RREB1	CATGCTCACACACACTGGTC	CGTGAGGTGAGGTCTAGCAC
RSP18	AGTTCCAGCATATTTTGCGAG	CTCTTGGTGAGGTCAATGTC
SERPIN1	CTCATCAGCCACTGGAAAGGCA	GACTCGTGAAGTCAGCCTGAAAC
SMAD3	CATGGACGCAGGTTCTCCAA	GGCTCGCAGTAGGTAACTGG
SNAI1	CGAGTGGTTCTTCTGCGCTA	GGGCTGCTGGAAGGTAAACT
TGFB1	TACCTGAACCCGTGTTGCTCTC	GTTGCTGAGGTATCGCCAGGAA
TP53	GTTCCGAGAGCTGAATGAGG	TCTGAGTCAGGCCCTTCTGT
XPC	TTGTCGTGGAGAAGCGGTCTAC	CTTCTCCAAGCCTCACCACTCT

2. Supplementary Figures

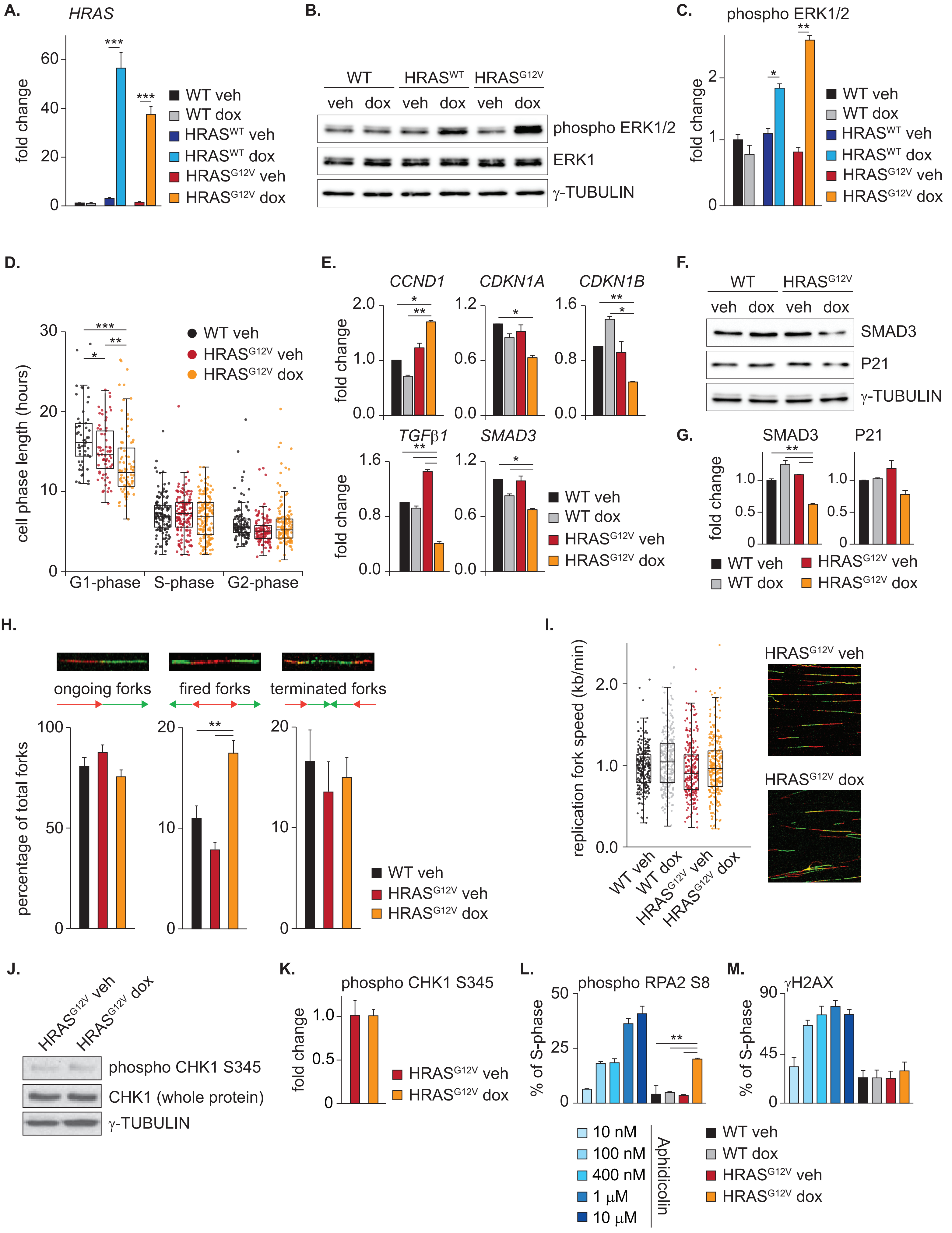

Supplemental Figure 1, related to Figure 1

Figure S1. Related to Figure 1

A Quantitative PCR of HRAS 24 hours after doxycycline administration in RPE cells with inducible $HRAS^{WT}$ or $HRAS^{G12V}$. Note the small increase in HRAS levels in RPE $HRAS^{G12V}$ vehicle cells. This is due to leakiness of the Tet repressor system and can result in a mild phenotype under certain conditions, e.g. in figure S1D. Statistical differences were evaluated using a Kruskal-Wallis test and post-hoc Dunnett's test.

B Representative immunoblot showing moderate and elevated levels of phosphorylated ERK1/2 in cells which overexpress wild-type and oncogenic HRAS respectively.

C Quantification of n=3 immunoblots of which a representative example is shown in B. Statistical differences were evaluated using a Kruskal-Wallis test and post-hoc Dunnett's test.

D Dot plot showing the length of cell cycle phases in cells with or without oncogenic RAS measured using live cell imaging. 100 cells per condition were analyzed. Statistical differences were evaluated using a Kruskal-Wallis test and post-hoc Dunnett's test.

E Quantitative PCR of genes regulating the G1-S transition of indicated conditions which were harvested when confluency was reached. Statistical differences were evaluated using a Kruskal-Wallis test and post-hoc Dunnett's test.

F Representative immunoblot of the samples as described in E.

G Quantification of n=3 immunoblots of which a representative example is shown in F. Statistical differences were evaluated using a Kruskal-Wallis test and post-hoc Dunnett's test.

H Quantification and representative examples of different types of DNA replication forks observed in a DNA fiber assay in the absence of drugs. Bars represent mean ± s.e.m. of 3 independent experiments, per experiment at least 250 fibers were analyzed. Statistical differences were evaluated using a Kruskal-Wallis test and post-hoc Dunnett's test.

I Dot plot showing the replication fork speed of individual DNA tracks measured using a DNA fiber assay. Per condition at least 100 fibers were measured. Representative images of fibers are shown on the right.

J Representative immunoblot showing levels of phosphorylated CHK1 in the absence and presence of oncogenic RAS.

K Quantification of n=3 immunoblots of which a representative example is shown in H. Statistical differences were evaluated using a Kruskal-Wallis test and post-hoc Dunnett's test.

L Quantification of n=2 flowcytometry experiments. Only S-phase cells were selected, based on DNA content, for analysis. Percentage of phospho RPA2-S8 positive S-phase cells was determined based on an unstained control. Cells were collected after 24 hours treatment with doxycycline or 16 hours treatment with Aphidicolin. Statistical differences were evaluated using a Kruskal-Wallis test and post-hoc Dunnett's test.

M Same as in L, but now with an antibody against γH2AX.

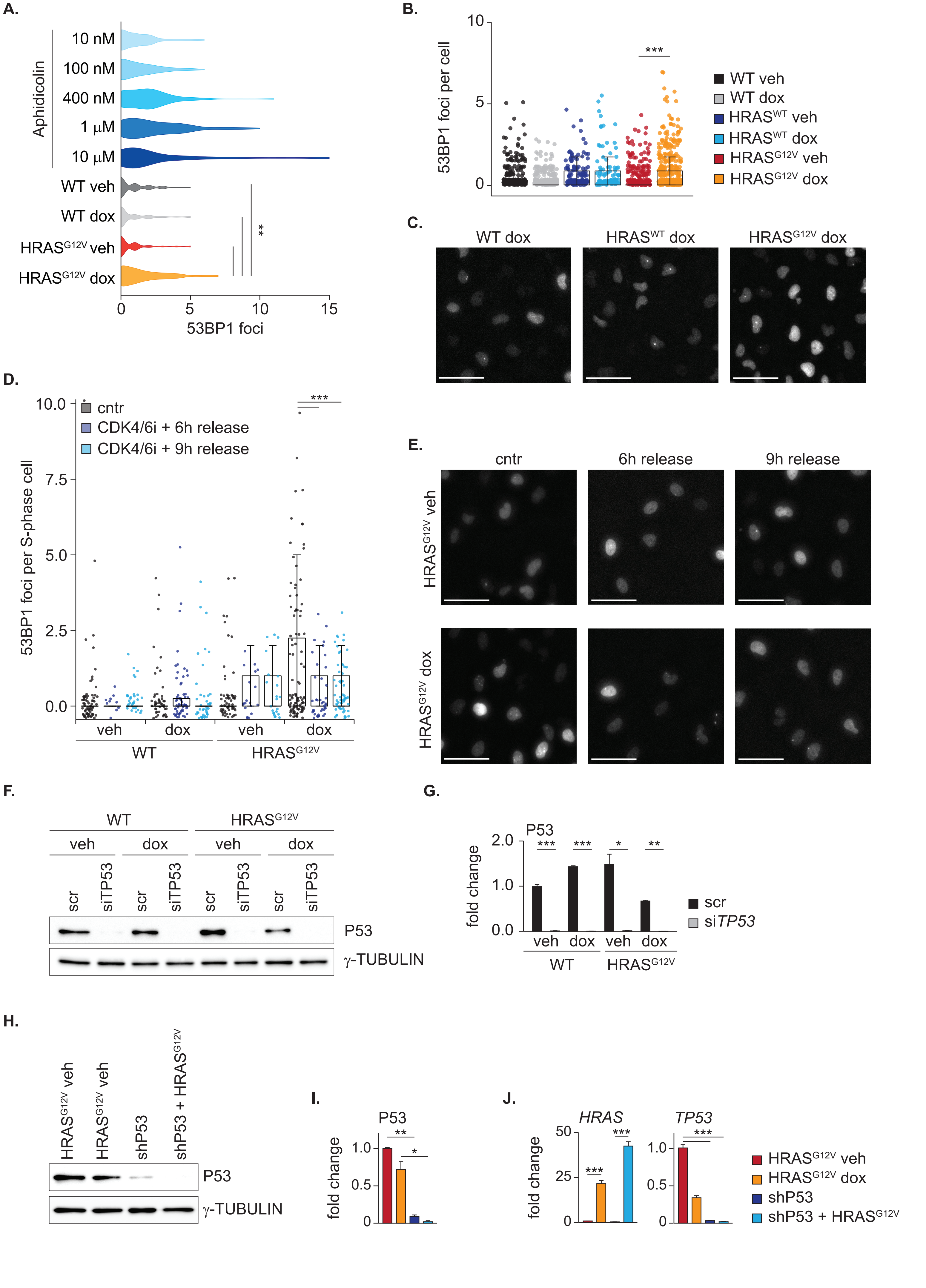

Supplemental Figure 2, related to Figure 1

Figure S2. Related to Figure 1

A Violin plot showing the number of 53BP1 foci per S-phase cell in indicated cell lines 24 hours after addition of doxycycline or in RPE wild-type cells 16 hours after treatment with Aphidicolin. Statistical differences were evaluated using a Kruskal-Wallis test and post-hoc Dunnett's test.

B Dot plot showing the number of 53BP1 foci, as read out for RS, per S-phase cell in the absence or presence of 24 hours wild-type or oncogenic RAS induction. 100 cells per condition were analyzed. Differences were statistically tested using a Kruskal-Wallis test with post-hoc Dunnett's test with Benjamin Hochberg correction.

C Representative images of the experiment described in B. Scale bar represents 50 μm.

D Dot plot showing the number of 53BP1 foci per cell in S-phase after indicated time of release from 24 hours CDK4/6 inhibitor treatment or in control, non-CDK4/6 inhibitor released, S-phase cells. Differences were statistically tested using a Kruskal-Wallis test with post-hoc Dunnett's test with Benjamin Hochberg correction.

E Representative images of the experiment described in D. Scale bar represents 50 μm.

F Representative immunoblot showing efficient depletion of P53 24 hours after treatment with si*TP53*.

G Quantification of n=3 immunoblots of which an representative example is shown in F. Statistical differences were evaluated using a Kruskal-Wallis test and post-hoc Dunnett's test.

H Representative immunoblot showing depletion of P53 in RPE cells stably expressing a short hairpin against P53.

I Quantification of n=3 immunoblots of which an representative example is shown in F. Statistical differences were evaluated using a Kruskal-Wallis test and post-hoc Dunnett's test.

J Quantitative PCR of HRAS and TP53 24 hours after doxycycline administration in RPE cells with inducible $HRAS^{G12V}$ or that stably express shP53. Statistical differences were evaluated using a Kruskal-Wallis test and post-hoc Dunnett's test.

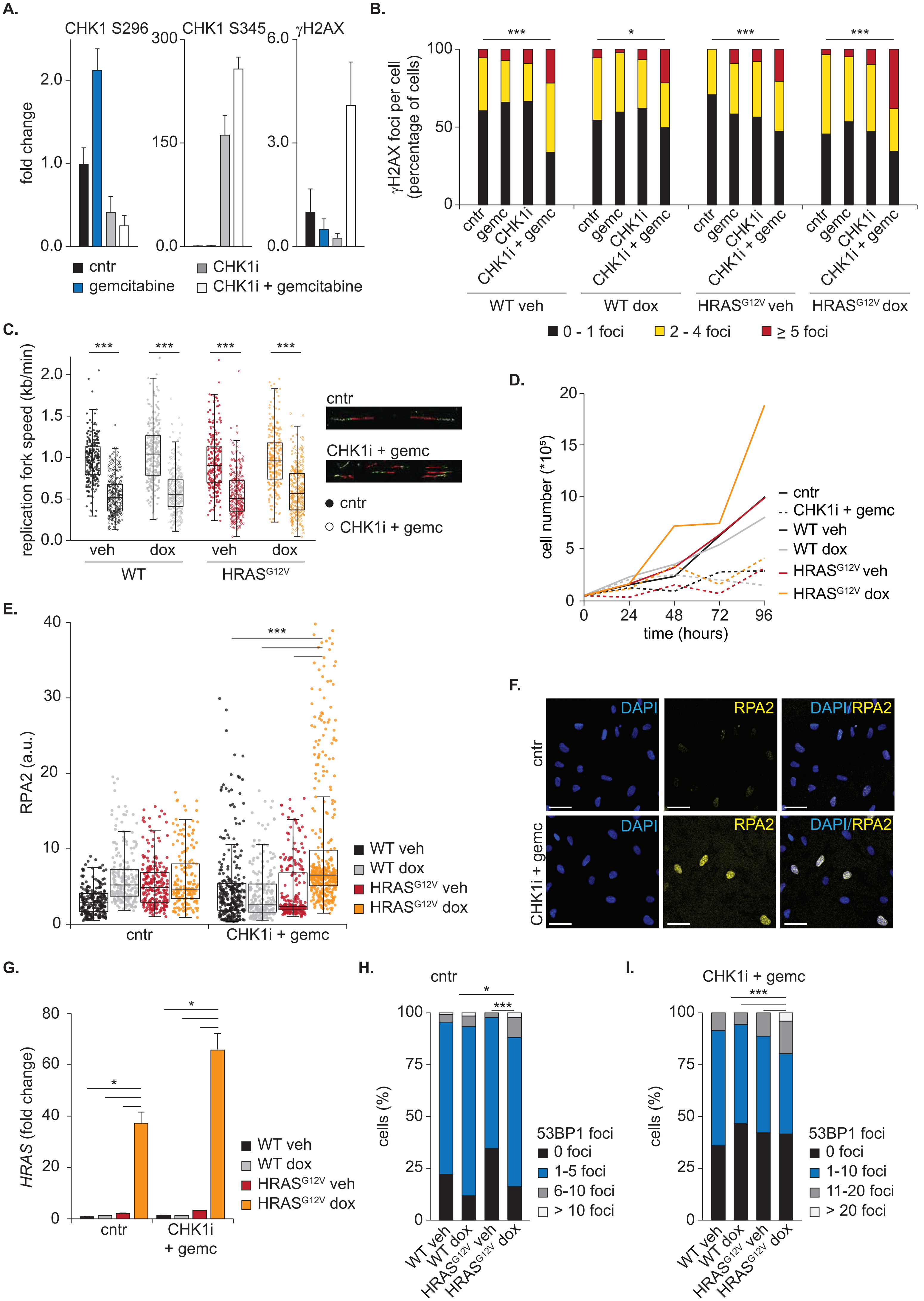

Supplemental Figure 3, related to Figure 2

Figure S3. Related to Figure 2

A Quantification of n=3 immunoblots of which a representative example is shown in Figure 2A. Statistical differences were evaluated using a Kruskal-Wallis test and post-hoc Dunnett's test.

B Bar chart showing the number of γH2AX foci, as read out for DNA damage, per cell in the absence or presence of oncogenic RAS and indicated drug treatment. At least 100 cells per condition were analyzed. Statistical differences were evaluated with a Chi-Square test corrected for multiple comparisons.

C Dot plot showing the replication fork speed of individual DNA tracks, before and 16 hours after treatment with CHK1i + gemcitabine, measured using a DNA fiber assay. Per condition at least 250 fibers were measured. Statistical differences were evaluated using a Student's t-test.

D Cell proliferation, as measured by cell counting, of RPE WT or $HRAS^{G12V}$ cells with or without doxycycline and CHK1i + gemcitabine.

E Quantification of immunofluorescence staining of DNA bound RPA2 in RPE cells before and 24 hours after treatment with CHK1i + gemcitabine. At least 100 cells per condition were evaluated. Differences were statistically tested using a Kruskal-Wallis test with post-hoc Dunnett's test with Benjamin Hochberg correction.

F Representative images of the experiment described in E. Scale bar represents 50 μm.

G Quantitative PCR of HRAS 24 hours after doxycycline administration in U2OS cells with inducible $HRAS^{G12V}$ in the absence and presence of CHK1i + gemcitabine. Differences were statistically tested using a Kruskal-Wallis test with post-hoc Dunnett's test with Benjamin Hochberg correction.

H Quantification of immunofluorescence staining of 53BP1 in U2OS cells. Bar chart shows the number of 53BP1 foci, as read out for RS, in individual cells in the absence or presence of oncogenic RAS. At least 100 cells per condition were analyzed. Statistical differences were evaluated with a Chi-Square test corrected for multiple comparisons.

I Same as H, but now in the presence of CHK1i + gemcitabine.

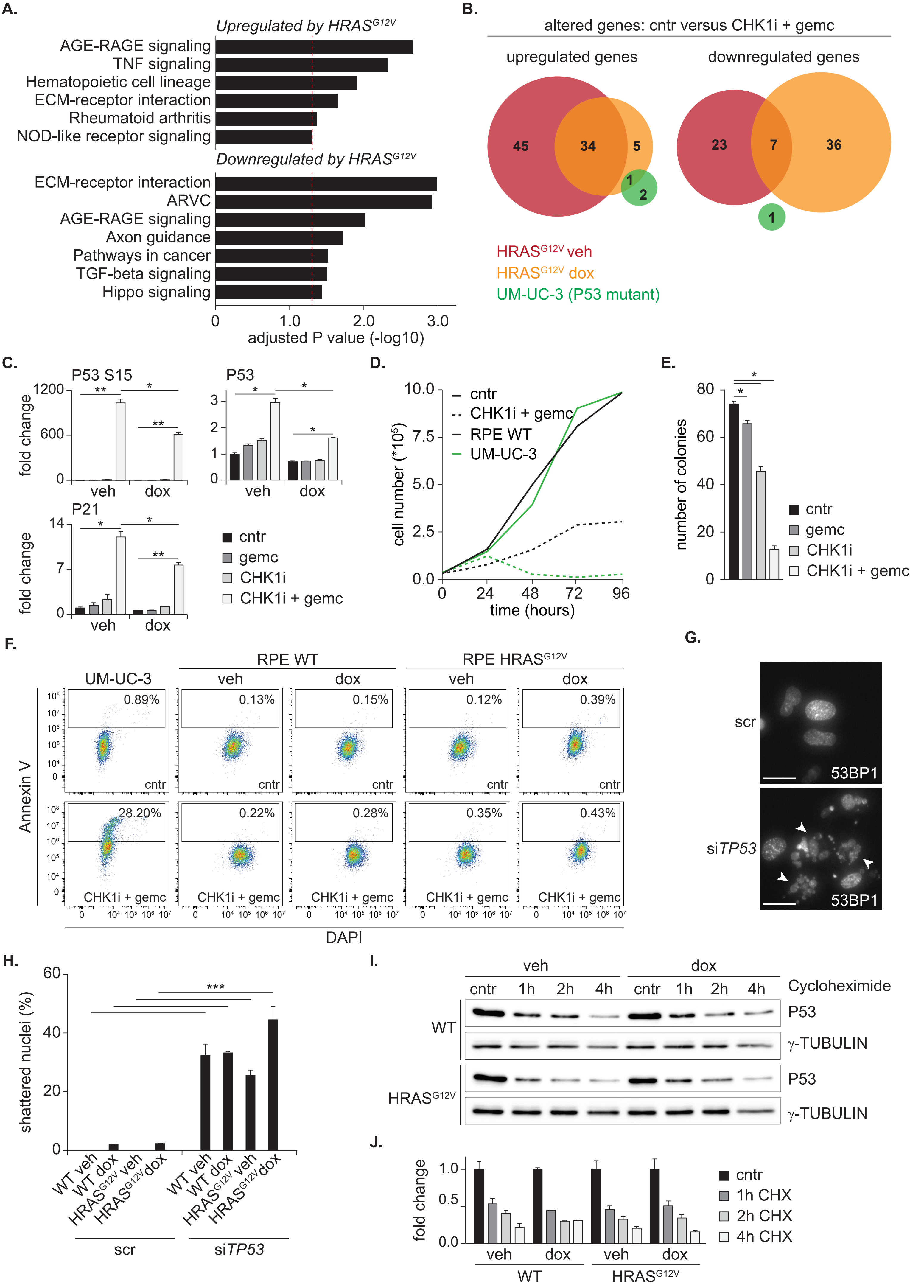

Supplemental Figure 4, related to Figure 3

Figure S4. Related to Figure 3

A Pathway analysis of differentially expressed, up or downregulated, genes in S-phase cells with (dox) and without (veh) $HRAS^{G12V}$. Significantly changed genes with a fold change of at least 1.5 were selected for analysis. Red dotted line indicates *P* value of 0.05.

B Venn diagrams of significantly changed genes, with a fold change of at least 1.5 before and after treatment with CHK1i + gemcitabine. RPE $HRAS^{G12V}$ S-phase cells with or without doxycycline and UM-UC-3 S/G2-phase cells were analyzed.

C Quantification of n=3 immunoblots of which a representative example is shown in Figure 3B. Statistical differences were evaluated using a Kruskal-Wallis test and post-hoc Dunnett's test.

D Cell proliferation, as measured by cell counting, of RPE WT and UM-UC-3 cells with or without CHK1i + gemcitabine.

E Quantification of colony formation assays. Cells were treated for 48 hours with indicated drugs and fixed after a recovery period of 10 days. Bars represent mean (sum of 3 technical replicates, mean of 3 independent experiments) ± s.e.m., statistical analysis was performed with a Kruskal-Wallis test.

F Annexin V staining analyzed by flow cytometry of indicated cell lines before and 48 hours after treatment with CHK1i + gemcitabine. Annexin V and DAPI positive cells indicate apoptotic cells.

G Representative example of nuclei 48 hours after treatment with CHK1i + gemcitabine in the presence or absence of P53. Arrowheads indicate shattered nuclei. Scale bar represents 20 μm.

H Quantification of shattered nuclei 48 hours after treatment with CHK1i + gemcitabine in the presence or absence of P53. At least 100 cells per condition were analyzed. Statistical differences were evaluated with a Chi-Square test corrected for multiple comparisons.

I Representative immunoblot of P53 in indicated cell lines before and after treatment with cycloheximide.

J Quantification of n=3 immunoblots of which a representative example is shown in I. Protein levels are normalized to the 0h timepoint of each individual cell line.

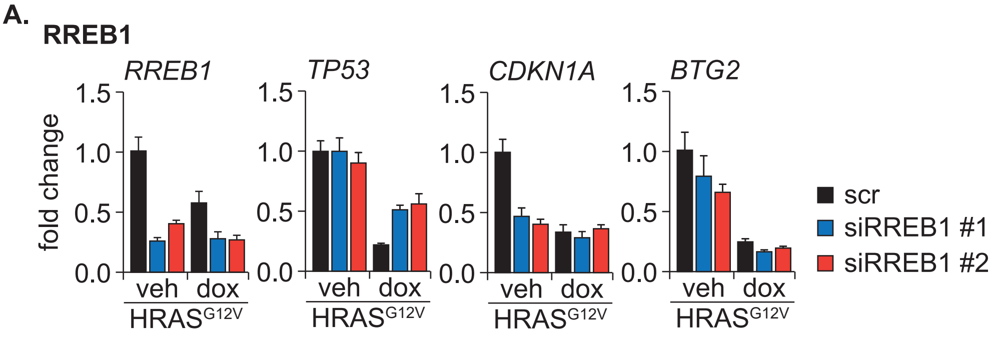

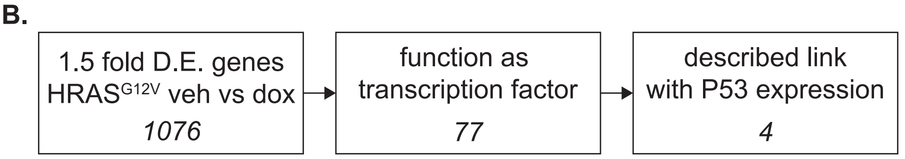

C.

putative transcriptional regulators of P53

gene	function	fold change cntr	fold change CHK1i + gemc
CEBPβ	repressor	2.55	3.58
KLF4	repressor	1.68	1.54
SMAD3	activator	- 1.61	- 1.60
KLF9	activator	- 2.78	- 2.97

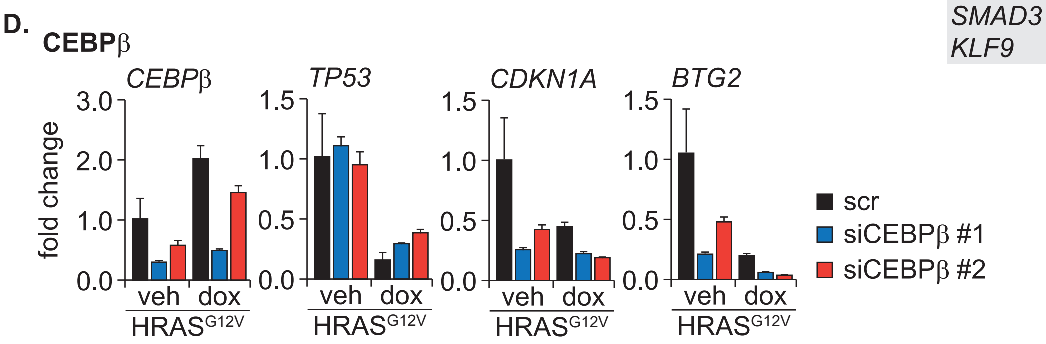

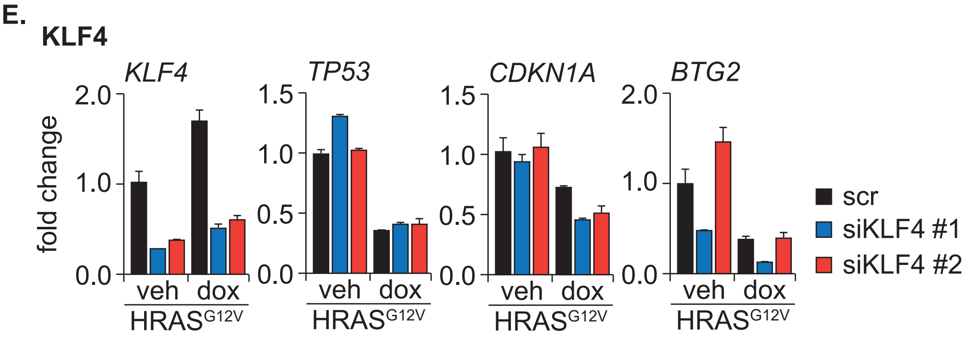

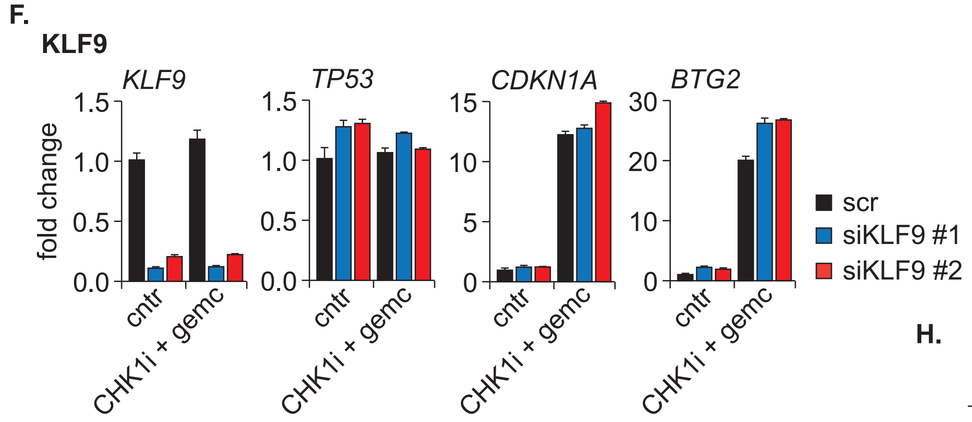

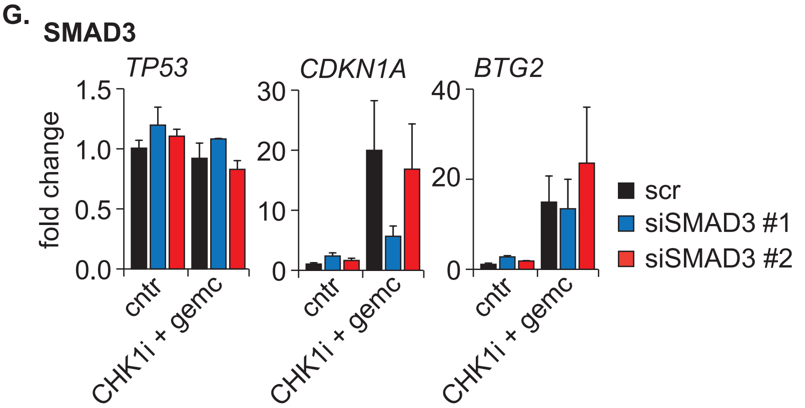

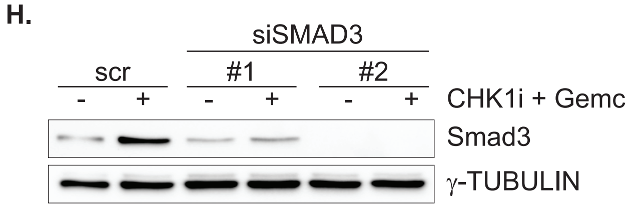

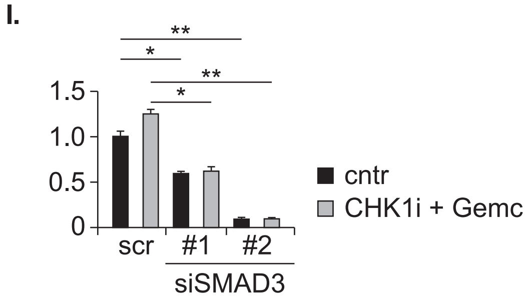

Supplemental Figure 5, related to Figure 4

Figure S5. Related to Figure 4

A Quantitative PCR of siRNA target (*RREB1*), *TP53* and P53 targets 24 hours after transfection of RPE $HRAS^{G12V}$ cells in the presence or absence of doxycycline. Bars represent mean ± s.e.m. of 2 independent experiments. Statistical differences were evaluated using a Kruskal-Wallis test and post-hoc Dunnett's test.

B Workflow describing how potential transcriptional regulators shown in C were selected from RNA-sequencing dataset.

C Table with putative P53 transcriptional regulators selected based on RNA-sequencing data. Fold changes, on a linear scale, between RPE cells with and without $HRAS^{G12V}$ in the absence (cntr) and presence of RS-inducing drugs (CHK1 + gemc) as detected using RNA-sequencing are shown.

D Same as A, but know with siRNA targeting *CEBP–beta*.

E Same as A, but know with siRNA targeting *KLF4*.

F Quantitative PCR of siRNA target (*KLF9*), *TP53* and P53 targets 24 hours after transfection of RPE WT cells in the presence or absence of CHK1i + gemcitabine. Bars represent mean ± s.e.m. of 2 independent experiments. Statistical differences were evaluated using a Kruskal-Wallis test and post-hoc Dunnett's test.

G Same as G, but know with siRNA targeting *SMAD3*.

H Representative immunoblot showing efficient knock down of SMAD3 in RPE cells 24 hours after transfection.

I Quantification of n=3 immunoblots of which a representative example is shown in H. Statistical differences were evaluated using a Kruskal-Wallis test and post-hoc Dunnett's test.

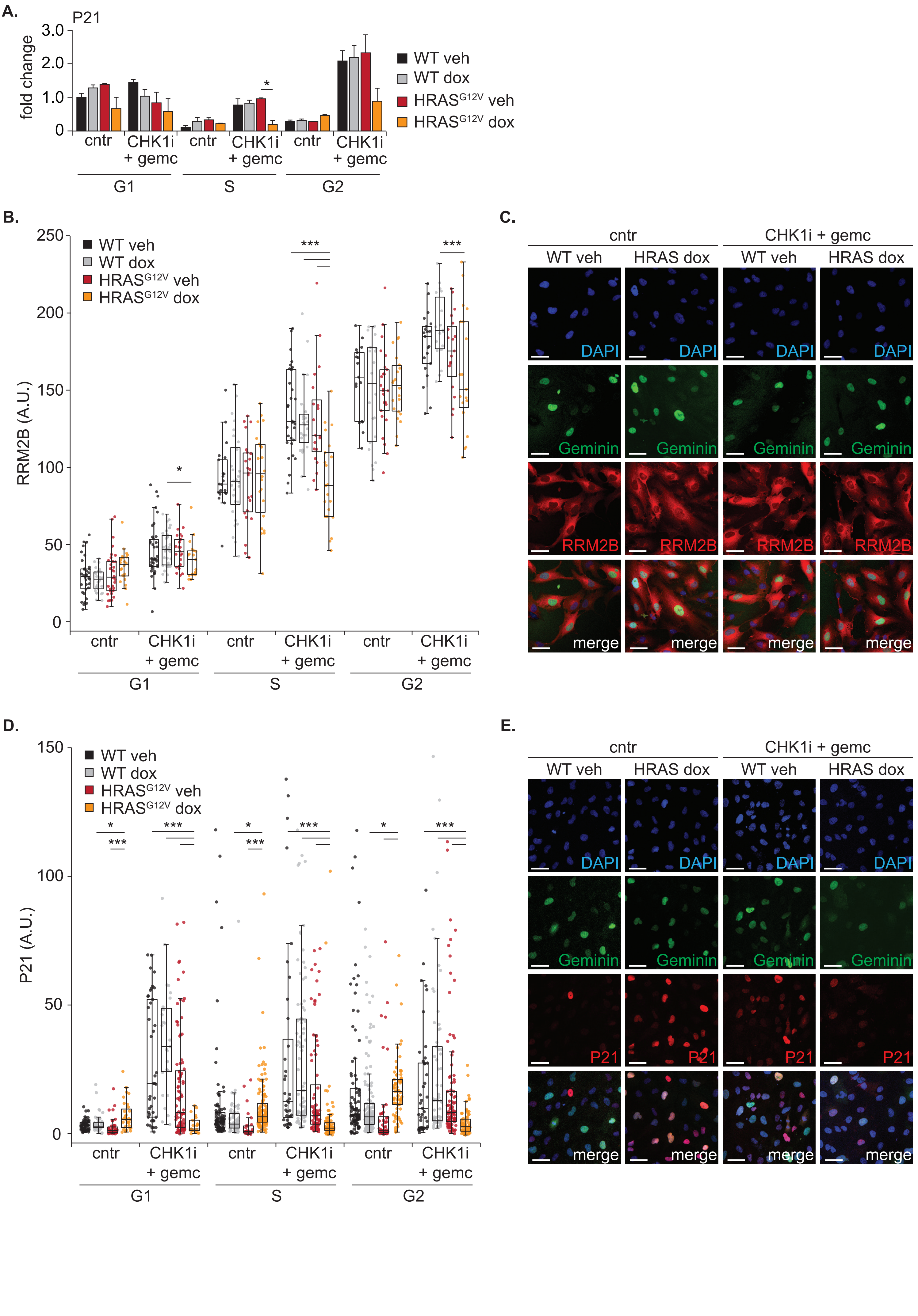

Supplemental Figure 6, related to Figure 4

Figure S6. Related to Figure 4

A Quantification of n=3 immunoblots of which a representative example is shown in Figure 4F. Statistical differences were evaluated using a Kruskal-Wallis test and post-hoc Dunnett's test.

B Dot plot showing a quantification of RRM2B immunofluorescence staining of cells 16 hours after treatment with CHK1i + gemcitabine. Cell cycle status was inferred from FUCCI4 cell cycle markers. At least 50 cells per condition were evaluated. Statistical differences were evaluated using a Kruskal-Wallis test and post-hoc Dunnett's test.

C Representative images of immunofluorescence staining of which the quantification is shown in B. Scale bar represents 50 μm.

D Dot plot showing a quantification of P21 immunofluorescence staining of cells 16 hours after treatment with CHK1i + gemcitabine. Cell cycle status was inferred from FUCCI4 cell cycle markers. At least 50 cells per condition were evaluated. Statistical differences were evaluated using a Kruskal-Wallis test and post-hoc Dunnett's test.

E Representative images of immunofluorescence staining of which the quantification is shown in D. Scale bar represents 50 μm.

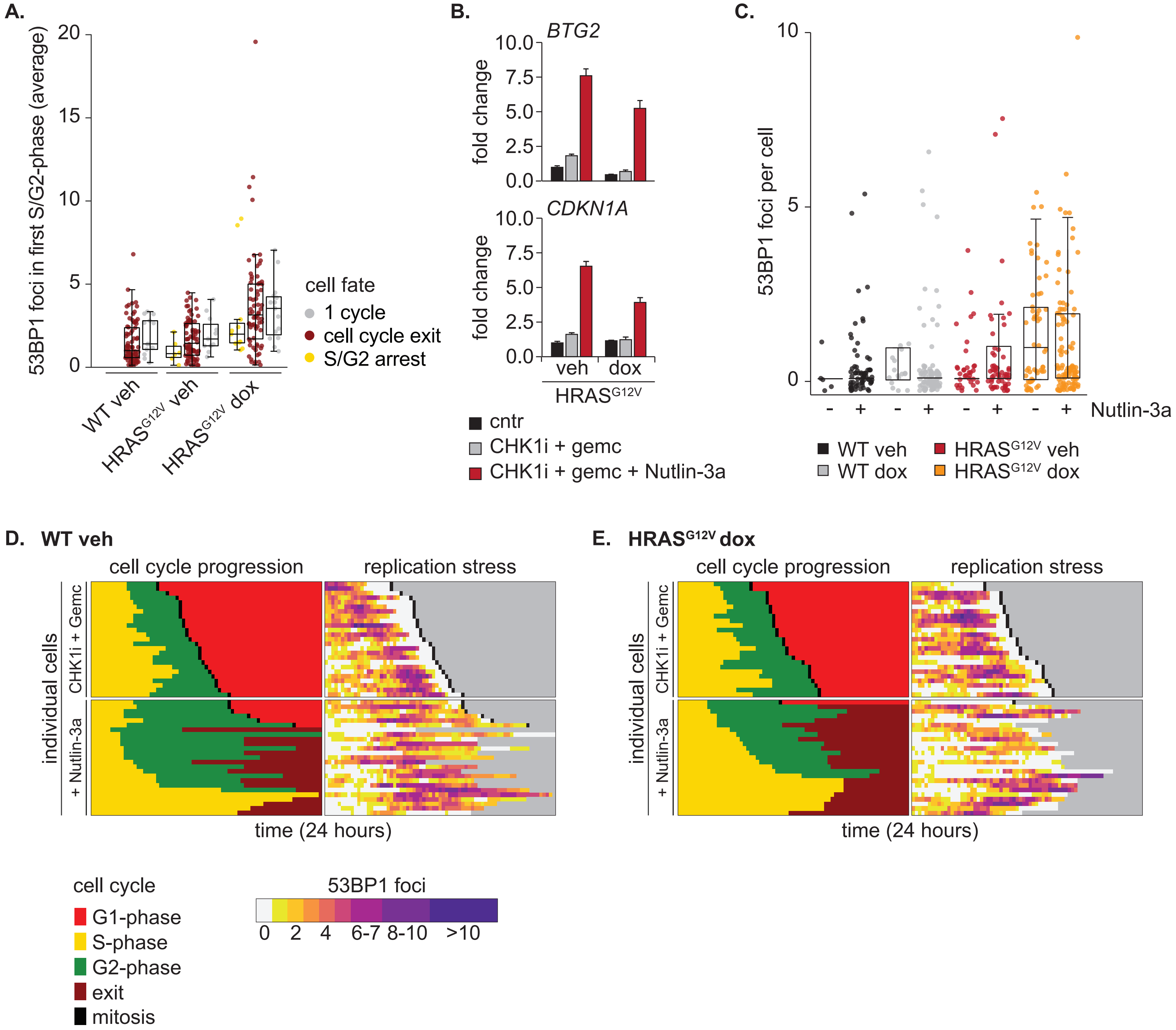

Supplemental Figure 7, related to Figure 5 and 6

Figure S7. Related to Figure 5 and 6

A Quantification of live cell imaging data showing the average number of 53BP1 foci per image per cell in S/G2-phase. Cells were separated based on their fate as shown in Figure 6B.

B Quantitative PCR showing an increase in P53 target gene expression when RPE cells are treated with Nutlin-3a in addition to CHK1i + gemcitabine. Cells were harvested 24 hours after treatment. Bars represent mean ± s.e.m. of 2 independent experiments. Statistical differences were evaluated using a Kruskal-Wallis test and post-hoc Dunnett's test.

C Dot plot showing the number of 53BP1 foci per cell in S-phase in indicated cell lines before and 24 hours after treatment with Nutlin-3a. Statistical differences were evaluated using a Kruskal-Wallis test and post-hoc Dunnett's test.

D Heatmap showing cell cycle progression and 53B1 foci in RPE WT cells which were in S-phase at the start of imaging. Cells were traced until they completed mitosis or exited the cell cycle.

E Same as D, but now with RPE HRAS^{G12V} dox cells.
